# Nitric oxide mediated transcriptional modulation enhances plant adaptive responses to arsenic stress

**DOI:** 10.1038/s41598-017-03923-2

**Published:** 2017-06-15

**Authors:** Pradyumna Kumar Singh, Yuvraj Indoliya, Abhisekh Singh Chauhan, Surendra Pratap Singh, Amit Pal Singh, Sanjay Dwivedi, Rudra Deo Tripathi, Debasis Chakrabarty

**Affiliations:** 1 Council of Scientific and Industrial Research - National Botanical Research Institute (CSIR-NBRI), Rana Pratap Marg, Lucknow, 226001 India; 2 Academy of Scientific and Innovative Research (AcSIR), Anusandhan Bhawan, 2 Rafi Marg, New Delhi, 110 001 India

## Abstract

Arsenic (As) contamination in rice leads to yield decline and causes carcinogenic risk to human health. Although the role of nitric oxide (NO) in reducing As toxicity is known, NO-mediated genetic modulation in the plant during arsenic toxicity has not yet been established. We analyzed the key components of NO metabolism and the correlations between NO interaction and arsenic stress using rice as a relevant model plant. Illumina sequencing was used to investigate the NO-mediated genome-wide temporal transcriptomic modulation in rice root upon AsIII exposure during 12 days (d) of the growth period. Sodium nitroprusside (SNP) was used as NO donor. SNP supplementation resulted in marked decrease in ROS, cell death and As accumulation during AsIII stress. NO was found to modulate metal transporters particularly NIP, NRAMP, ABC and iron transporters, stress related genes such as CytP450, GSTs, GRXs, TFs, amino acid, hormone(s), signaling and secondary metabolism genes involved in As detoxification. We detected NO-mediated change in jasmonic acid (JA) content during AsIII stress. The study infers that NO reduces AsIII toxicity through modulating regulatory networks involved in As detoxification and JA biosynthesis.

## Introduction

Arsenic (As) contamination in water has been an issue of grave concern in South East Asian countries, causing health risks for an estimated 65 million people^1^. Irrigation of crops with As-contaminated water results in accumulation of As in food crops such as rice, wheat and vegetables^2–4^. Nearly half of the world population is dependent on rice as the main diet. As-contamination in rice is a major problem in terms of food safety and human health risk^5, 6^.

Among the two inorganic forms of As, AsIII is more toxic than AsV, and the latter is found predominantly in the paddy field during water logged conditions. To minimize As induced stress, plants have several adaptive mechanisms, such as reduction in As uptake and efflux from root cells, ROS scavenging activity, synthesis of glutathione (GSH) and binding of AsIII with phytochelatins (PCs) and sequestration of As into the vacuole^3, 7–9^. Recent studies revealed that nitric oxide (NO) plays a significant role in the reduction of As toxicity by regulating antioxidant defense systems that efficiently regulate oxidative stress^10, 11^.

Nitric oxide is an important endogenous signaling molecule involved in a variety of plant functions such as plant growth and development, and responses *vis-a-vis* environmental stresses^12^. Crosstalk between NO and phytohormones, such as auxin, cytokinin, ethylene, abscisic acid (ABA), emphasized the role of NO in different physiological processes^13^. NO is also involved in primary root growth and development by regulating the cytokinin level, auxin transport and signaling^14, 15^. Being an antioxidant, NO reduces ROS content and prevents cellular damage by quenching superoxide radical^16^. The enzymes of Ascorbate- glutathione cycle are potential targets for nitration and S-nitrosylation by NO-post translational modification modulating enzyme activities^17, 18^. Exogenous application of NO decreases As uptake, enhances the expression of gamma-glutamylcysteine synthetase to increase the GSH content and maintains the ratio of GSH/GSSG in plants^11, 19^. Glutathione react with NO and forms S-nitrosoglutathione (GSNO) which acts as a reservoir of NO in cells and regulates the expression of transcription factors such as WRKY and MYB, involved in plant response to pathogens and abiotic stress^20, 21^. Previous studies indicate that NO modulates the expression of genes encoding, ABC-transporters, cytochrome P450, glutathione peroxidase, glutathione S-transferase, glutaredoxins, signal transduction, pathogen resistance and cell death in the *Arabidopsis* plants^21, 22^. Overproduction of NO up-regulated the expression of genes involved in drought and salt stress tolerance and enhanced proline content in rice^23^. However, the molecular basis of NO function during arsenic toxicity is poorly understood. Moreover, the relationship between As stress and NO interaction, and molecular mechanisms of NO-mediated alleviation of As stress are still not known. The present study was therefore carried out to analyze the key components of NO metabolism (genome-wide transcriptomic analysis) and also to strengthen our understanding of the interrelationships between NO interaction and arsenic stress using rice as a relevant model crop.

## Results and Discussion

### Growth parameter and As analysis

**Figure 1:**
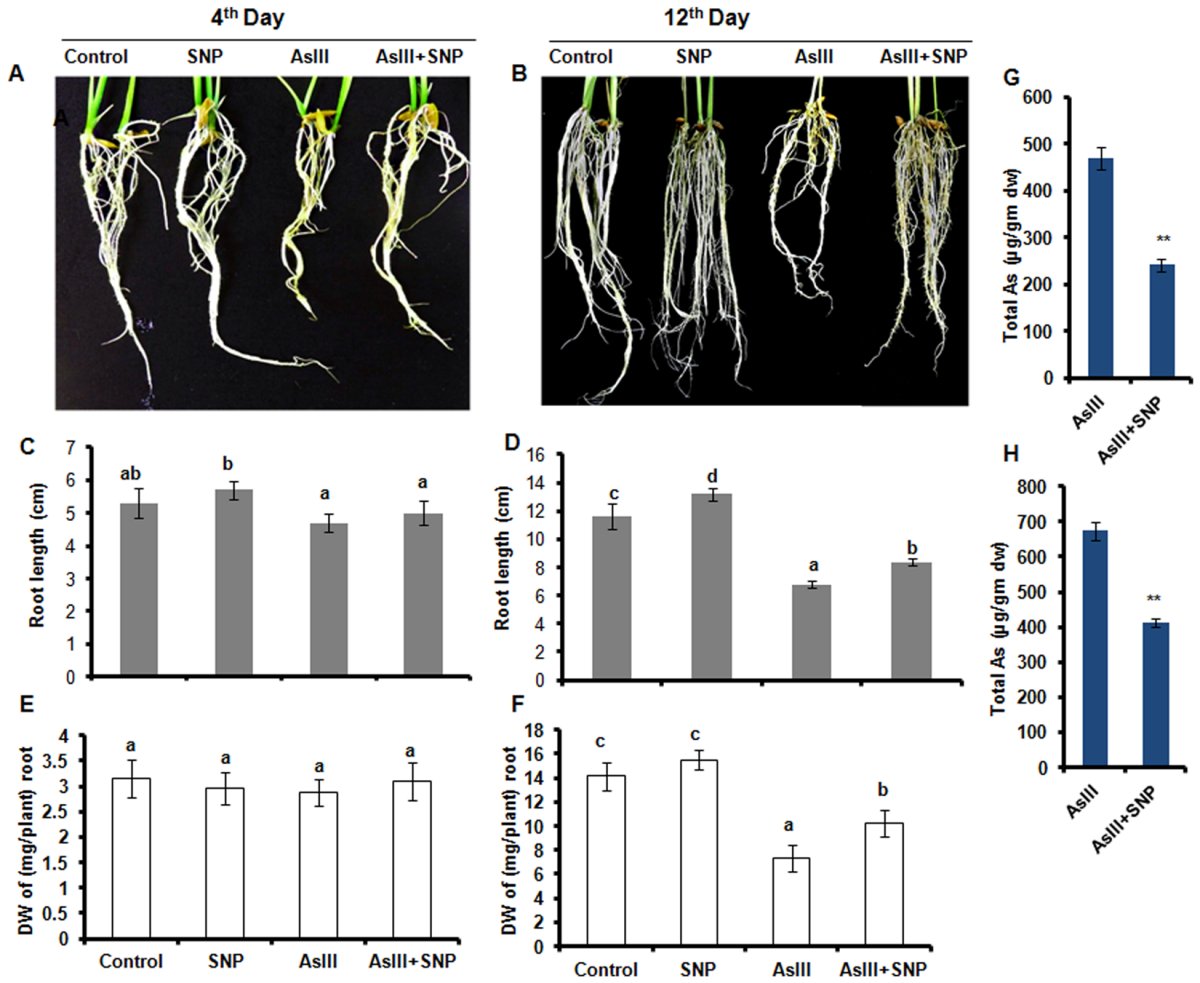
Morphological observation of roots on 4^th^ day (**A**) and 12^th^ day (**B**), Growth parameter measurement (root length) on 4^th^ day (**C**), and 12^th^ day (**D**). The dry weight of roots on 4^th^ day (**E**) and 12^th^ day (**F**) indicated a significant increase in dry weight of the AsIII + SNP treated root as compared to the AsIII treated root on 12^th^ day. Arsenic accumulation in root on 4^th^ day (**G**) and 12^th^ day (**H**) showed a significant decrease in As accumulation in the AsIII + SNP treatment as compared to the AsIII treatment.

The present study examined molecular signaling of NO and adaptive responses of the plant to cope with arsenic stress conditions. Rice seedlings treated with 25 μM AsIII resulted in ~11% and ~41% decline in root length on 4^th^ and 12^th^ day, respectively. While supplementation of 30 μM SNP along with 25 μM AsIII, showed 5% and 27% decline in root length on day 4^th^ and 12^th^, respectively, in comparison to the control (Fig. 1A,B). The result indicated that the root experienced higher As toxicity with increasing time exposure (Fig. 1C,D). No significant difference in roots length was found in all treatment in comparison to the control on 4^th^ day (Fig. 1C). However, a significant increase in dry weight was observed on 12^th^ day in the AsIII + SNP treated root in comparison to the AsIII treated root (Fig. 1F). Arsenic stress resulted in a significant increase in As contents in roots after both 4^th^ and 12^th^ day of treatment. Supplementation of SNP (NO donor) along with AsIII stress remarkably reduced As contents in roots of rice seedlings (Fig. 1G,H). Rice seedlings were grown under 25 μM AsIII treatments accumulated ~469 and ~674 µg/g dry weight of As in root on day 4^th^ and 12^th^, respectively. However, with SNP (AsIII + SNP) supplementation, the metalloid accumulation decreased significantly, recording 242 and 413 µg/g dry weight of As in root on day 4^th^ and 12^th^, respectively (Fig. 1G,H). NO-mediated reduction in As accumulation was consistent with plant growth as reported previously^11^. Interestingly, NO-mediated reduction in As accumulation decreased with a time interval, as a reduction in total As content (~48% on 4 day and ~38% on 12^th^ day) in the AsIII + SNP treated root was more on 4^th^ day compared to 12^th^ day.

### Detection of NO, superoxide radical (O_2_^·−^) and cell viability assay

**Figure 2:**
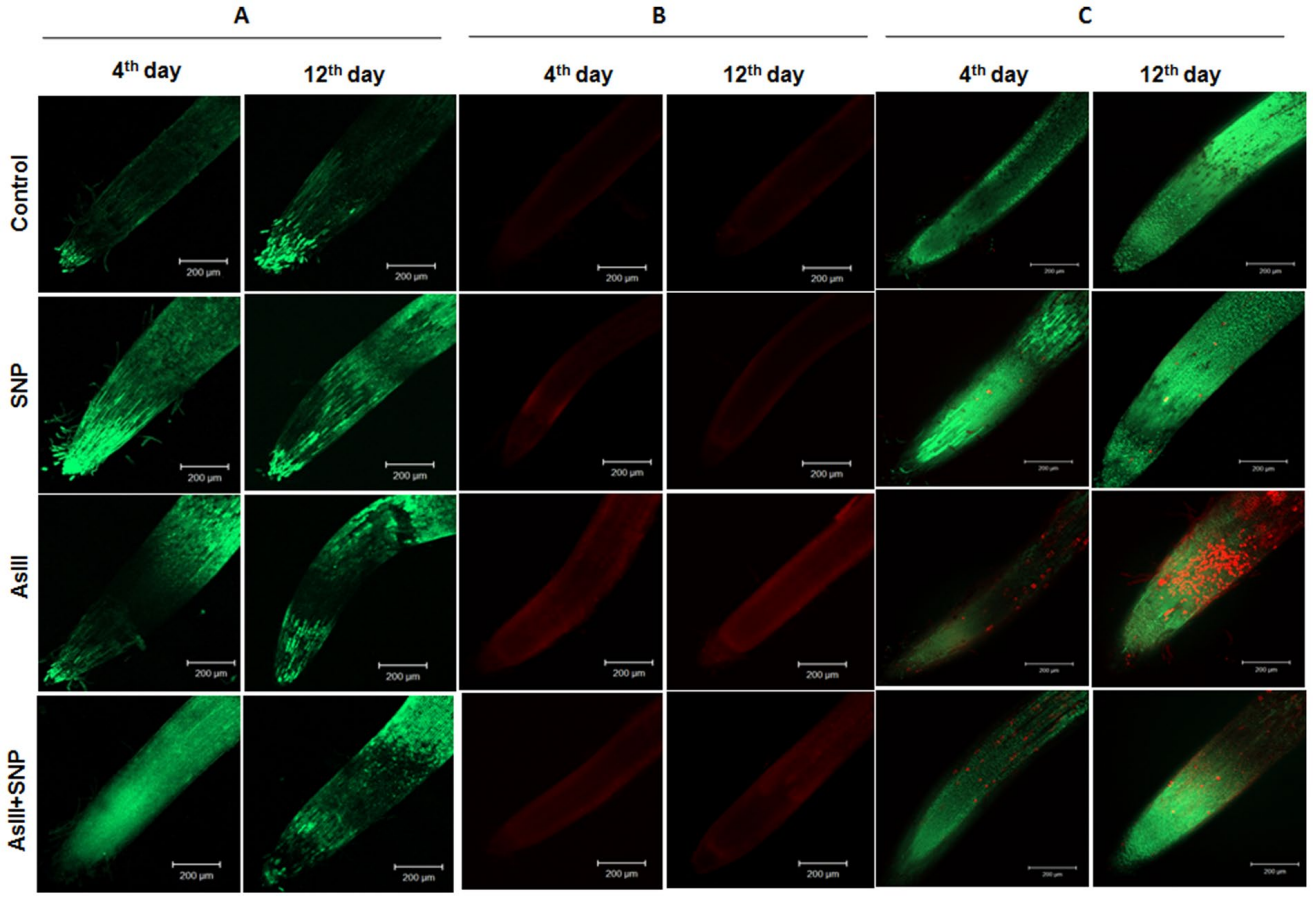
CLSM detection of NO, superoxide and cell viability. Green fluorescence (**A**) represented NO level in different treatments on 4^th^ and 12^th^ day. Red fluorescence (**B**) showed superoxide content in various samples at both time intervals. Cell viability (**C**) assay indicated viable and dead cell in green and red fluorescence respectively on 4^th^ and 12^th^ day. Results showed higher NO level, cell viability and lower superoxide radical content in the AsIII + SNP treated root as compared to the AsIII treated root.

Endogenous level of NO in rice root increased in all treatments than control at both exposure durations (Fig. 2A, Supplementary Figs 1 and 3A). DHE staining indicated decreased superoxide level in the SNP supplemented (AsIII + SNP) root as compared to the AsIII treatment at both exposure durations (Fig. 2B, Supplementary Figs 2 and 3B). Furthermore, cell viability was enhanced in the AsIII + SNP exposed root as compared to the AsIII treated root on both exposure durations (Fig. 2C, Supplementary Figs 4 and 5). The As accumulation in the 4^th^ day AsIII and 12^th^ day AsIII + SNP treated root was almost same, but As toxicity was more in 12^th^ day AsIII + SNP treatment in comparison to 4^th^ day AsIII treatment (Supplementary Figs 4 and 5). We also found that the level of superoxide was less in 12^th^ day AsIII + SNP treated root in comparison to day 4^th^ AsIII treated root (Supplementary Fig. 3B). Our results demonstrated that NO maintained cell viability and decreased superoxide content and cell death. Superoxide is a strong oxidizing agent that negatively affects membrane integrity of the cell^24^. Being an antioxidant, NO can directly bind with superoxide radical and convert it into ONOO^−^ (peroxynitrite) which is less stable in cellular environments^16^. Moreover, NO-mediated reduction in As content may also be associated with a reduction of toxicity, resulting in increased cell viability.

### Nitric oxide modulates transcriptional profiling under AsIII stress

**Figure 3:**
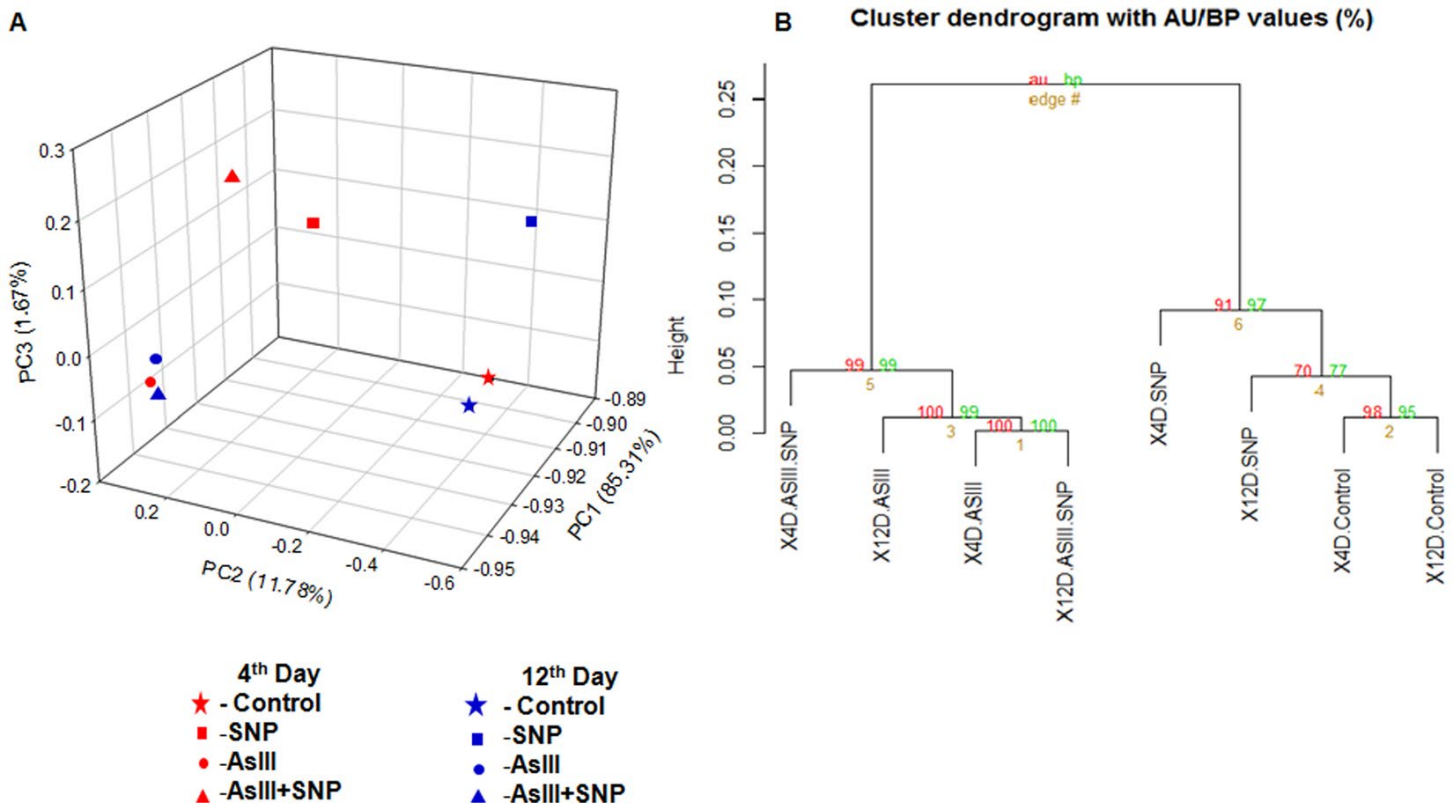
Global transcriptomic correlations among different treatments at both time durations. PCA (**A**) and hierarchal clustering (**B**) showed less variation between control and SNP while significant variation was observed between the control and AsIII + SNP treatment of day 4^th^ and 12^th^ day. Expression level was calculated in different samples using FPKM value.

Previous studies reported that NO supplementation reduced As accumulation to cope with As toxicity^10, 11, 25^. In the present study, we analyzed key components of NO and molecular networks under arsenic stress using rice as the model plant. The Illumina Hiseq 2000 sequencing platform (2 × 100 bp) was used to generate the high-quality data (Supplementary Fig. 6). To analyze the global expression pattern of genes under different treatments and time intervals, Principal Component Analysis (PCA) was performed. The least variation in expression pattern was found between control and SNP treatment (Fig. 3A) at both exposure durations. Interestingly, the expression pattern of 4^th^ day AsIII and 12^th^ day AsIII + SNP treated roots was quite similar. However, 4^th^ day AsIII + SNP and 12^th^ day AsIII treated root showed different expression pattern. The transcriptional differences seem to decrease adverse effect of AsIII, as SNP supplementation showed the protective role *vis-a-vis* AsIII toxicity. PCA data is also in agreement with the phenotypic data, described earlier, where AsIII compared to the AsIII + SNP treated root showed more toxicity (Fig. 1A,B). PCA was further validated using the hierarchical clustering algorithm (Fig. 3B).

**Figure 4:**
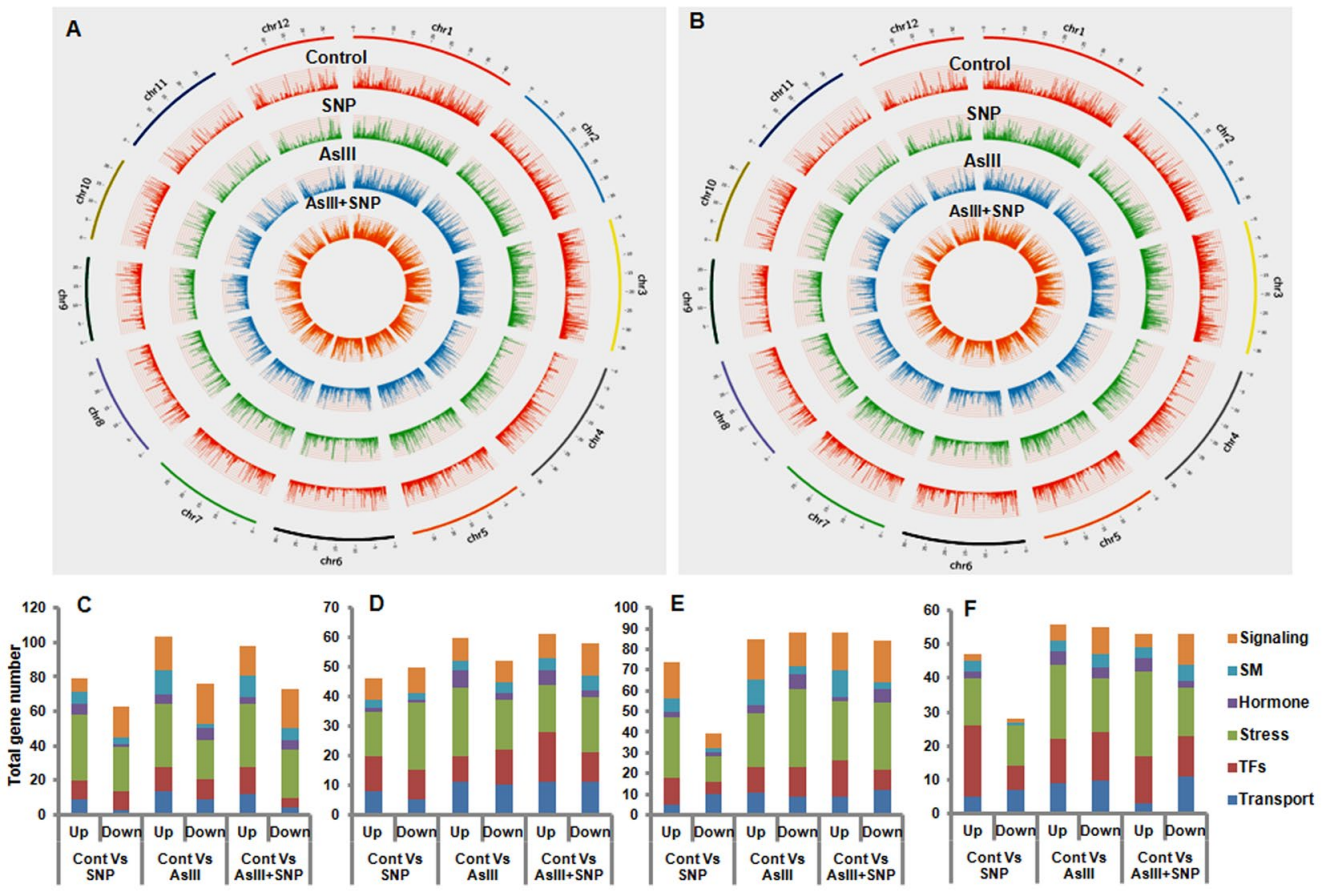
Expressions patterns of globally expressed genes at different chromosomes were analyzed by using expression (FPKM) value on 4^th^ (**A**) and 12^th^ day (**B**), respectively. Total gene number of signaling, hormone, stress, TFs, transporter and secondary metabolism (SM) related genes (**C**) on 11, (**D**) on 12 chromosomes at 4^th^ day and (**E**,**F**) on 11 and 12 chromosomes respectively at 12^th^ day. Results showed enrichment (relative expression) of stress related genes on 11 and 12 chromosomes in the AsIII + SNP treatment among analyzed gene sets.

The Circos analysis was performed to elucidate the visual snap shot of differences in the expression pattern of globally expressed genes at every chromosome in different treatments (Fig. 4A,B). Circos analysis revealed that relative transcript abundance in the AsIII + SNP treated root was lower than AsIII treated root (except chromosome no. 3, 5, 7, 9 and 10) on 4^th^ day (Supplementary Fig. 7A). In contrast, relative transcript abundance in the AsIII + SNP exposed root was higher than AsIII treated root on each chromosome (except chromosome no. 9) on 12^th^ day (Supplementary Fig. 7B). However, the total number of genes expressed in the AsIII + SNP treatment was higher than the AsIII treatment on each chromosome on 4^th^ day (Supplementary Fig. 7C). However, it was lower in the AsIII + SNP treatment in comparison to AsIII treatment on each chromosome on 12^th^ day (Supplementary Fig. 7D). Interestingly, on day 4^th^, the relative abundance of transcript (Total FPKM) at chromosome 12 was much higher in AsIII than the AsIII + SNP treatment, but the total number of genes expressed was more in the AsIII + SNP treatment. However, the inference on abundance of transcripts and a total number of expressed genes was found *vice versa* on day 12^th^. It appeared that chromosome 12 is more responsive during the AsIII + SNP treatment. We also analyzed the different set of genes related to stress, signaling, TFs, transporter, hormone and secondary metabolism expressed on chromosome 11 and 12, to find out their relative response in AsIII + SNP stress (Fig. 4C–F). Stress-related genes were more responsive on 11 and 12 chromosomes at both exposure durations. Signaling and TFs related genes showed significant expression variation on 12^th^ day (Fig. 4E,F). It suggested that stress-related genes were more enriched (relative expression) in the AsIII + SNP treatment among analyzed gene sets. Abiotic stress related genes present on chromosome 12 such as Cyt P450, GSTs were also found to be modulated during As stress^26–28^. Contrasting behavior of relative abundance at chromosome 12 among AsIII and AsIII + SNP treated roots at both exposure durations might be due to complex genomic dynamics at low (on day 4^th^) to higher As toxicity (on day 12^th^). Prolonged AsIII exposure leads to increased cell lethality, thereby negatively affecting transcript expression.

A total of 1959 and 1692 genes were differentially expressed (p ≤ 0.05, up or down-regulation of genes ≥  ± log 2 fold change occur at least in one comparative sample) in exposed samples compared to control on 4^th^ and 12^th^ day, respectively (Supplementary Table 1). Venn analysis was performed to identify the differentially up-regulated and down-regulated genes among different samples. In all samples, up and down-regulation of differentially expressed genes (DEGs) were analyzed with respect to the control. The total numbers of DEGs were higher in the AsIII + SNP treated root as compared to AsIII treated roots on day 4^th^ (Supplementary Fig. 8A,B). Interestingly, on 12^th^ day, a total number of down-regulated DEGs were less (584) in the AsIII + SNP in comparison to the AsIII treated root (635) (Supplementary Fig. 8C,D). The difference in number of DEGs in the AsIII and AsIII + SNP treated samples indicated an important role of NO in the modulation of gene expression. The transcriptomic difference was further validated using qRT PCR (Supplementary Figs 9,10,11 and 12). Two internal controls were used to validate the RNA-Seq data. The expression patterns of genes were matched with RNA-Seq data.

### Nitric oxide modulated expression behavior of genes (K-means clustering)

K-means clustering was performed to find out the relative expression pattern of different sets of genes related to the transporter, transcription factors, hormone and secondary metabolisms in different treatments at both exposure durations. DEGs were divided into five clusters on the basis of their expression patterns (Supplementary Fig. 13A,B; Supplementary Table 2). On 4^th^ day, 46 transporters genes of cluster I was more up-regulated in the AsIII + SNP treatment than in the AsIII and SNP treatments. Furthermore, cluster II showed 46 genes of transporters which showed higher expression in the AsIII treatment in comparison to SNP and AsIII + SNP treatments. Similar expression pattern was observed in cluster I, II, and IV on 12^th^ day. In general, most of the transporter genes showed lower expression in the AsIII + SNP treatment in comparison to the AsIII treatment on 4^th^ day, but on day 12^th^, it was higher in the AsIII + SNP treatment as compared to the AsIII treatment (Supplementary Fig. 13B). Transcription factors also showed differential expression patterns at both exposure durations. A total of 32 transcription factor genes in cluster II on 4^th^ day, and 45 transcription factor genes on 12^th^ day were more up-regulated in the AsIII + SNP treatment in comparison to the SNP and AsIII treatments. While genes of cluster III and cluster I on 4^th^ and 12^th^ day respectively, showed lower expression in the AsIII + SNP treatment than SNP and AsIII treatments. Briefly, transcription factors also showed lower expression in the AsIII + SNP treatment in comparison to the AsIII treatment on 4^th^ day but showed opposite expression in the AsIII + SNP treatment as compared to AsIII treatment on 12^th^ day. Interestingly, hormone metabolism genes in cluster II represent the genes which showed less expression in AsIII treatment in comparison to SNP and AsIII + SNP treatments. On 12^th^ day, cluster III of hormone metabolism genes showed higher expression in the AsIII + SNP treatment compared to SNP and AsIII treatments. Clustering of secondary metabolism genes indicated higher expression in the AsIII + SNP treatment in comparison to SNP and AsIII treatments on 4^th^ day. Results demonstrated that the expression patterns of various set of genes were entirely different on 4^th^ and 12^th^ day and indicated NO-mediated transcriptional modulation occurred with time duration.

### Pathway analysis

Differential trancriptomic dynamics was carried out to explore detailed molecular and signaling cascades in the NO-mediated reduction of As content and toxicity. In this study, we focused on genes of pathways, such as metal accumulation and transportation, amino acid, lipid and hormone metabolism, transcriptional regulation and stress signaling, where the majority of those were found modulated by NO under AsIII stress. The pageman analysis was used to annotate the DEGs. The DEGs were classified on the basis of their biological function such as stress, lipid, hormone and secondary metabolism, signaling, transport, transcriptional regulation, etc. (Supplementary Table 1). A large set of genes were annotated as “not assigned” as the annotation of the rice genome does not assign any putative functions, and many genes were stated as “unspecified” or “unknown”.

### Nitric oxide modulated metal transporters under AsIII stress

**Figure 5:**
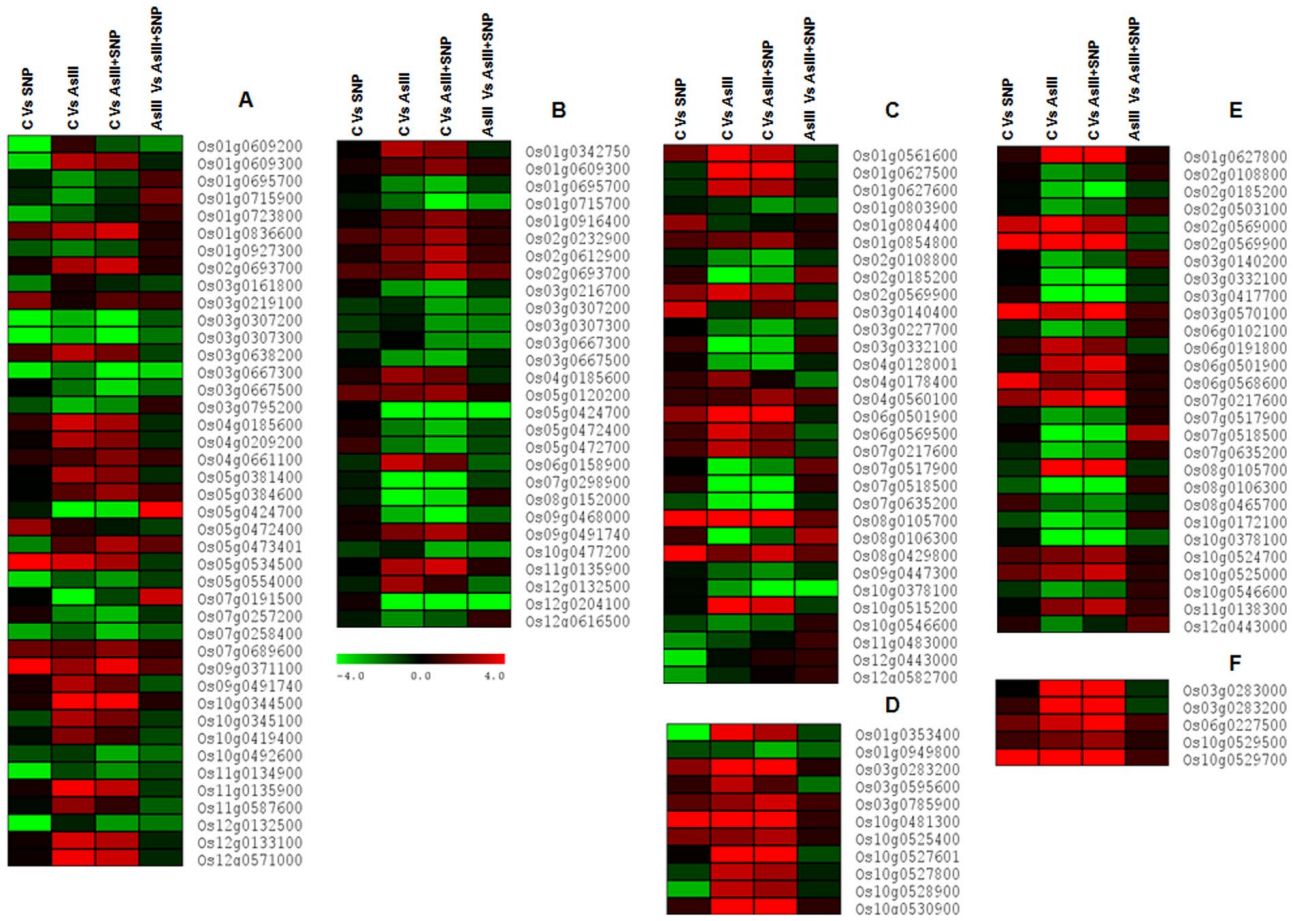
NO-mediated modulation of transporters and stress related genes. Differential expression patterns of NIP, ABCB, ABCC and ABCG transporter family proteins on 4^th^ day (**A**) and 12^th^ day (**B**) indicated their important role in AsIII detoxification. Expression behavior of Cyt P450 and GSTs were represented by (**C**,**D**) on 4^th^ day, while (**E**,**F**) represented the 12^th^ day. Several GSTs and peroxidase family genes showed up-regulation in the AsIII + SNP treatment indicated a protective role of NO in AsIII stress. Red and green color showed up and down-regulation of genes respectively.

To investigate the NO-mediated modulation of As uptake, detoxification-related genes were studied (Fig. 5A,B). On 4^th^ day, among the 1959 DEGs, 77 belonged to metal uptake, binding and transport (Supplementary Table 3). Nodulin 26-like intrinsic (NIP) aquaporin channels are key players for AsIII uptake and translocation. The genes of the NIP family such as Os08g0152000, Os02g0745100 (*OsNIP3;2*, *OsNIP2;1*) were down-regulated in the AsIII and AsIII + SNP treatments at both time durations. However, *OsNIP2;1* was more down-regulated in the AsIII + SNP treatment compared to the AsIII treatment on 4^th^ day. Interestingly, Os12g0204100 (*OsNIP3;5*) was highly down-regulated (16 fold) in the AsIII + SNP treatment compared to the AsIII treatment (6 fold) on 12^th^ day. It was noted that NO reduced the As accumulation at both exposure durations (Fig. 1G,H). It indicated that the NO-mediated modulation in the expression of aquaporins might be a strategy for lowering As uptake during AsIII stress. The genes Os04g0209200, Os11g0587600, and Os09g0332700 (*OsABCB5*, *OsABCC9*, *OsABCG48*, and *OsPDR20*) were more up-regulated in the AsIII treatment in comparison to the AsIII + SNP treatment at both exposure durations. On 12^th^ day, Os01g0695700 (*OsABCB4*) showed down-regulation in the AsIII + SNP treatment compared with other treatments. Os06g0158900 (*OsABCC15*) was down-regulated in SNP exposed roots, while 3.1 and 1.5 fold up-regulated in the AsIII and AsIII + SNP treatments, respectively, in comparison to control (Fig. 5B). Among metal binding, chelation and storage DEGs, the Os07g0689600 (*OsNAS3*) was up-regulated 2.1 fold in the AsIII + SNP treatment and 1.4 fold in AsIII treatment on 4^th^ day (Fig. 5A; Supplementary Table 3). The differential expression of AsIII + SNP responsive genes including the NIP, ABCB, ABCC and ABCG transporter family proteins indicated their direct or indirect role in AsIII detoxification through a reduction in As uptake, efflux from the cell and vacuolar sequestration^3, 26^. Interestingly, Fe transporter *OsPEZ1* was up-regulated in the AsIII + SNP treatment in comparison to control at both exposure durations. The Os07g0258400 and Os07g0257200 genes (*OsNRAMP1*, *OsNRAMP5*) were more down-regulated (3.1 and 2.9 fold) in the AsIII + SNP treatment as compared to the AsIII treatment (1.49 and 2.1 fold) on 4^th^ day. The previous study indicated that As accumulation also reduced the iron uptake^29^. *OsNRAMP* and *OsPEZ* play a key role in uptake and translocation of As, Fe and Cd^30–33^. It suggested that a complex mechanism is involved to maintain Fe homeostasis and to lower As content in the root, but the whole mechanism is still unclear and yet to be investigated.

### Nitric oxide modulated stress responsive gene expression under AsIII stress

To explore the possible role of NO in the alleviation of AsIII toxicity, we focused on expression patterns of stress responsive genes. The different sets of stress responsive genes such as glutathione S-transferases (GSTs), glutaredoxins (GRXs), cytochrome P450s, heat shock proteins (HSPs), and peroxidases (PODs), were found to be significantly modulated at both time intervals (Fig. 5C–F; Supplementary Table 4). Interestingly, most GSTs were more up-regulated in the AsIII + SNP treatment compared to the AsIII treatment at both exposure durations. The Os03g0283200 (*OsGSTL1*) and Os03g0283100 genes (*OsGSTL2*) were more up-regulated in the AsIII + SNP treatment on 4^th^ day in comparison to the control, indicated their key role in heavy metal tolerance and detoxification^34, 35^. Another set of stress-related genes involved in glutathione biochemistry such as *OsGRX9* (Os02g0618100) and *OsGRX4* (Os01g368900) were also significantly up-regulated in the AsIII + SNP treatment on 12^th^ day, suggesting their protective role during As stress. Recent studies also elucidated that the *OsGRX9* and *OsGRX4* genes rendered tolerance against AsIII toxicity by modulating aquaporins expression and maintained S-glutathionylation and GSH content^36, 37^. Higher expression of cytochrome P450 (Os08g0105700, Os01g0627400 and Os02g0569900) genes were observed in the AsIII and AsIII + SNP treatments at both time intervals. Moreover, most of the genes related to the peroxidase family were up-regulated in the AsIII + SNP treatment at both exposure durations (Supplementary Table 4). The three HSPs, *OsHSP17*, *OsHSP18*.*0-CII*, and *OsHSP26* (Os01g0136200, Os01g0184100 and Os03g0245800) genes were significantly up-regulated up to 18.2, 20.6 and 17.4 fold, respectively in the AsIII + SNP treatment, and 16.7, 19.2 and 15.6 fold, respectively in the AsIII treatment, in comparison to the control on 4^th^ day. Our results indicated that NO-mediated reduction in ROS content during AsIII stress (Fig. 2B), and that NO modulated expression behavior of stress related genes such as GSTs, GRXs, HSPs and PODs might be a strategy to cope with As toxicity by maintaining glutathione biochemistry and redox potential in the cell^26^.

### Nitric oxide modulated nitrogen, amino acid and hormone metabolism genes under AsIII stress

**Figure 6:**
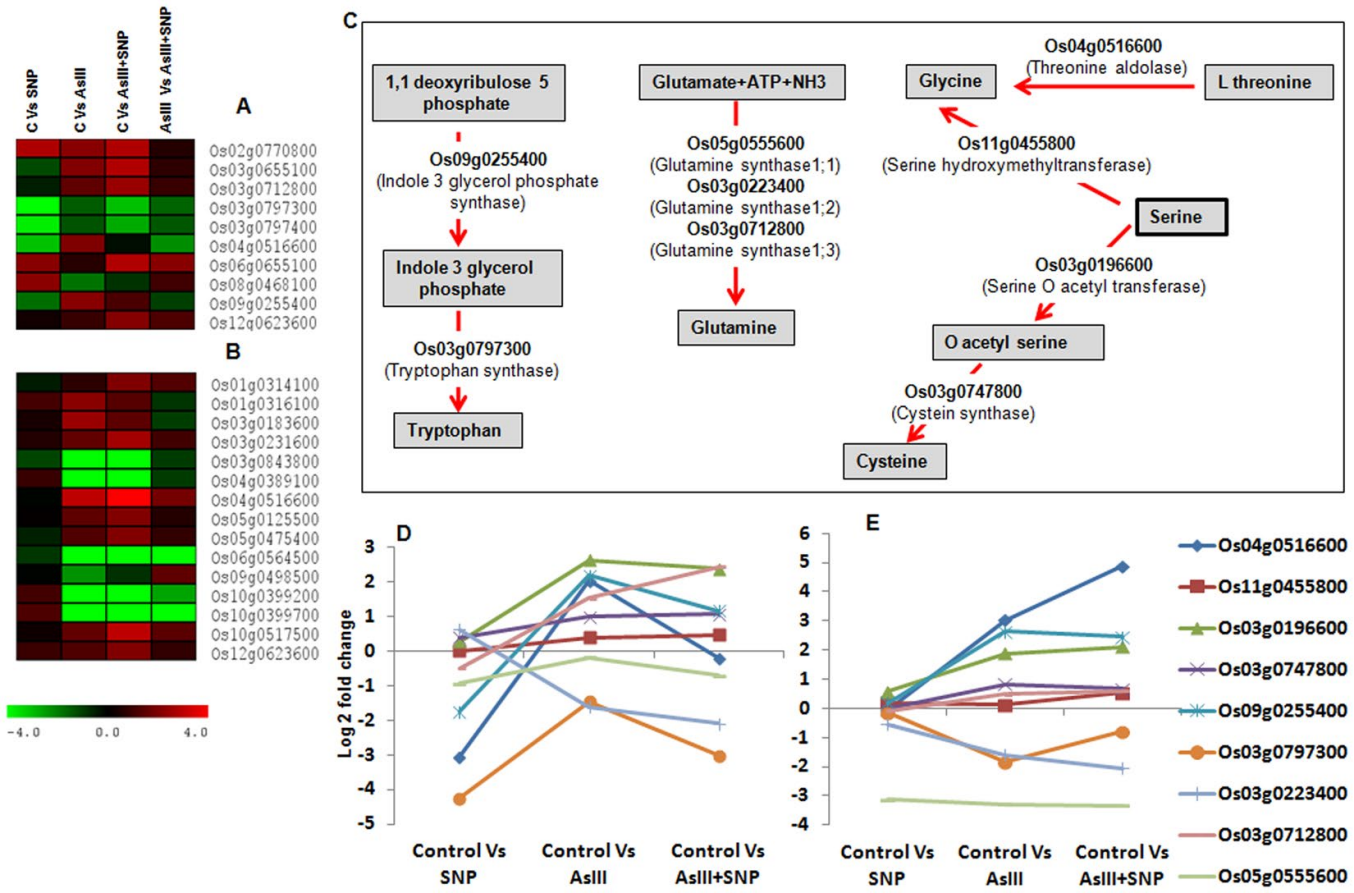
Differential expression analysis of amino acid and nitrogen metabolism related genes. (**A**,**B**) amino acid and nitrogen metabolism on 4^th^ and 12^th^ day respectively. (**C**) Modulation of the amino acid biosynthesis pathway (putative) and differential expressions of their candidate genes on 4^th^ day (**D**) and 12^th^ day (**E**). The modulation of amino acid and nitrogen metabolism gene in the AsIII and AsIII + SNP treatment suggested that NO might be involved in maintaining nitrogen metabolism during AsIII stress.

**Figure 7:**
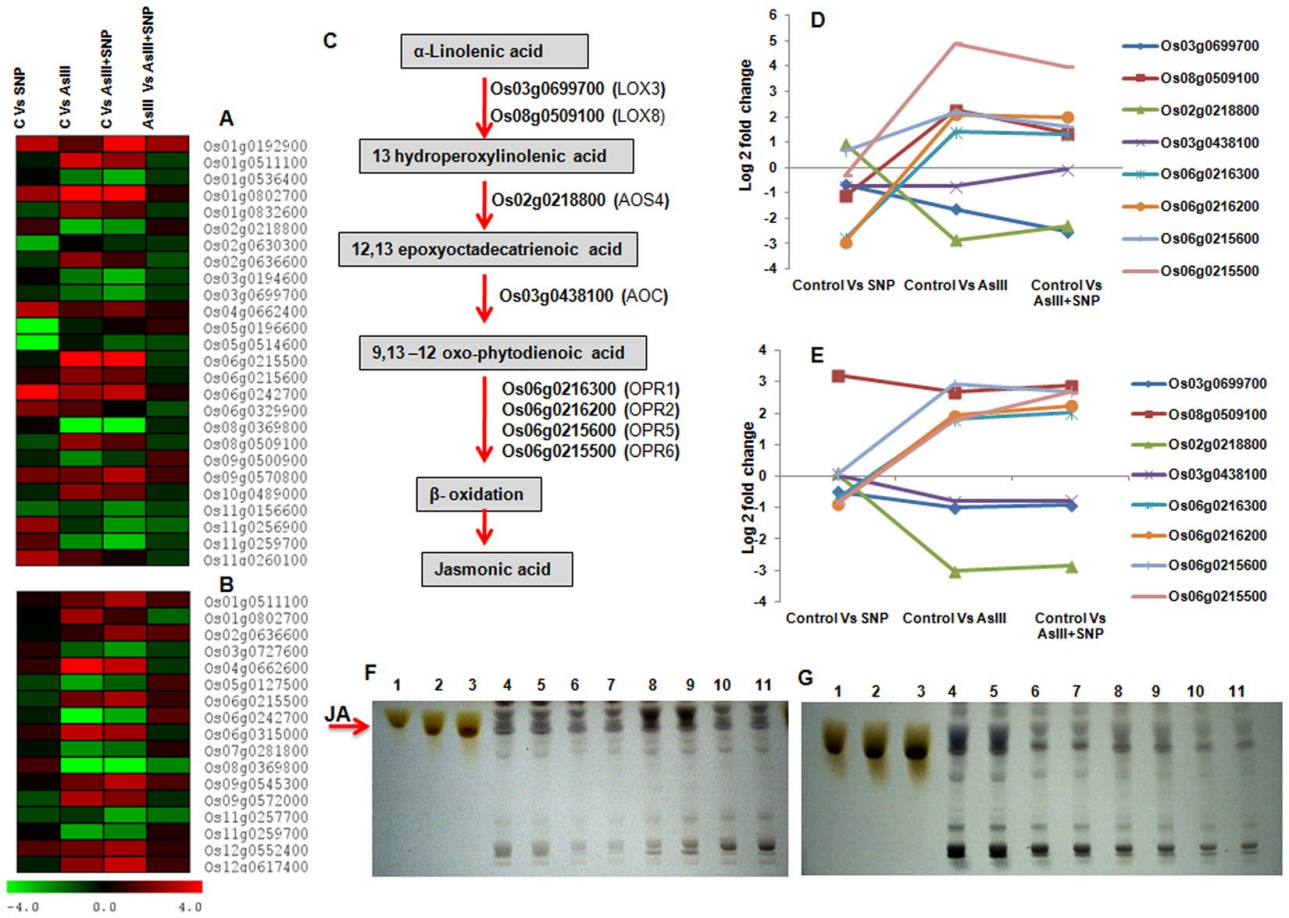
Comparative analysis of hormone metabolism related genes on 4^th^ day (**A**) and 12^th^ day (**B**). (**C**) Modulation of jasmonic acid (JA) biosynthesis pathway genes on 4^th^ day (**D**) and 12^th^ day (**E**). (**F**,**G**) JA quantification on 4^th^ and 12^th^ day respectively. Lane 1–3, JA standard; 4–5, control; 6–7, SNP; 8–9, AsIII treatment and 10–11, AsIII + SNP treatment.

Nitrogen and hormone metabolism, as a regulator of plant growth and development, is modulated by heavy metal toxicity and is tightly linked with amino acid and protein metabolism^29, 38^. We, therefore, focused on the analysis of genes involved in amino acid and nitrogen metabolism (Fig. 6A,B; Supplementary Table 5). The Os08g0468100 (*nitrate reductase*) gene was significantly up-regulated (2.1 fold) in the SNP treatment but down-regulated (1.8 fold) in the AsIII and (0.79 fold) AsIII + SNP treatments on 4^th^ day. The up-regulation of glutamine biosynthesis (Os03g0712800; *glutamine synthetase*) and nitrogen metabolism (Os02g0770800; *nitrate reductase*) genes (Fig. 6C,D) were observed in the AsIII + SNP treatment on 4^th^ day. It suggested that NO might be directly or indirectly associated with maintaining nitrogen assimilation and amino acid content during AsIII stress^39, 40^. Glycine and cysteine amino acid biosynthesis related genes Os04g0516600 (*L-allo-threonine aldolase*), and Os03g0196600 (*serine acetyltransferase*) were up-regulated up to 4.8 and 2.09 fold, respectively in the AsIII + SNP treatment, while 3 and 1.8 fold, respectively in the AsIII treatment, in comparison to the control on 12^th^ day (Fig. 6E). It may be a strategy to maintain cysteine and glycine pool in the AsIII + SNP treatment to cope with the As stress4. Up-regulation of tryptophan biosynthesis gene (Os09g0255400; *indole-3-glycerol phosphate synthase*) was found in the AsIII and AsIII + SNP treatments at both exposure durations (Fig. 7A,B; Supplementary Table 6). The gene of auxin metabolism, Os01g0802700 (*OsPIN9*) was more up-regulated in the AsIII + SNP as compared to other treatments on 4^th^ day. On day 12^th^, gene Os09g0545300 (*OsSAUR39*) related with auxin synthesis and transport was up-regulated up to 2.8 fold in the AsIII + SNP treatment in comparison to control. Further, NO promoted cell viability and root growth during AsIII stress (Figs 1 and 2). It suggested that NO might be involved maintaining auxin signaling, distribution and cell division during AsIII stress^41–43^. The cytokinin biosynthesis gene Os05g0311801 (*Isopentenyl transferase*: *IPT7*) was found to be up-regulated up to 5 fold in the AsIII and AsIII + SNP treatments at both time intervals as compared to control. Previous studies also indicated the role of cytokinin in the regulation of NO-mediated cell division and development^15, 44^. Results indicated that a complex regulatory cross-talk might be underway between NO, auxin and cytokinin during As stress^45^. Furthermore, gibberellins metabolism related gene Os03g0618300 (*gibberellin 20 oxidase 2*) was more up-regulated in the AsIII + SNP treatment compared to the AsIII treatment on 12^th^ day. A total of seven DEGs on 4^th^ day and five on 12^th^ day were found to be related to jasmonic acid (JA) metabolism (Fig. 7C). In general, the genes of the JA biosynthesis pathway were found to be more up-regulated in the AsIII treatment in comparison to the AsIII + SNP and SNP treatments (Fig. 7D,E). We also observed that SNP treatment reduced the JA content at both time intervals as compared to the control. Interestingly, the level of JA was higher in the AsIII treated root in comparison to SNP and AsIII + SNP treated roots at both durations (Fig. 7F,G; Supplementary Fig. 14A,B). Possible explanation for the reduction of the JA content in the AsIII + SNP treatment compared to the AsIII treatment may be due to a NO-mediated reduction in As toxicity^46^. The significant changes in the JA content suggest a crucial role of JA during AsIII stress, but the detailed mechanism of action of JA is unknown^27, 45, 47^.

### Nitric oxide modulated transcription factors under AsIII stress

Transcription factors (TFs) act as transcriptional regulators by modulating the gene expression and play an important role in abiotic stress. In this study, a total of 154 genes of transcriptional regulation were observed to be significantly express, in which 43 DEGs were common at both time intervals (Supplementary Fig. 15A,B; Supplementary Table 7). The Os02g0546600 and Os02g0521100 genes (*OsERF98* and *OsERF107*) were found to be up-regulated (>12 fold) in all treatments but up-regulation was higher in the AsIII + SNP treatment in comparison to the AsIII treatment on 12^th^ day. In this context, some transcription factors showed time-dependent expression patterns in treatments. For example, Os08g0454000 (*OsERF12*) and Os02g0546600 (*ERF98*) genes were differentially expressed only on 12^th^ day. A Recent study suggested the role of *OsERF* in ethylene biosynthesis and drought tolerance^48^, but their functional relation during NO and AsIII supplementations are not known. The genes (Os11g0303800, Os07g0497500, Os02g0168200, Os06g0112700 and Os02g0754400) of MYB TF family were more up-regulated in the AsIII + SNP treatment compared to the AsIII treatment on 4^th^ day. Interestingly, Os02g0168200 gene (*MYB* family transcription factor) was highly up-regulated (19.3 fold and 4.3 fold) in the AsIII + SNP treatment in comparison to the AsIII treatment at both exposure durations. In general, TFs belonging to the WRKY domain transcription factor family were more down-regulated in the AsIII treatment compared to the AsIII + SNP treatment on 4^th^ day and *vice versa* on 12^th^ day. Though, Os05g0528500 (*OsWRKY58*) and Os01g0586800 (*OsWRKY27*) genes were observed to be more up-regulated in the AsIII + SNP treatment in comparison to the AsIII treatment on 4^th^ and 12^th^ day respectively. Studies also highlighted the roles of the MYB and WRKY TFs family genes providing the tolerance against different stresses such as salt, drought, cold and biotic stress^49, 50^. The NO-mediated modulation in the expression of TFs indicated their extensive role in AsIII stress but their functional roles are yet to be investigated.

### Nitric oxide-mediated modulation of other pathway

The other pathway related genes such as lipid, secondary metabolism, xenobiotics degradation and signaling were also modulated by NO supplementation during AsIII stress (Supplementary Fig. 15C,D; Supplementary Table 8). A total of 47 genes on day 4^th^ and 45 genes on day 12^th^, related to secondary metabolisms were differentially expressed. Interestingly, the genes Os04g0178300 (*OsCYC1*), Os02g0571100 (*OsCYC2*), Os04g0179700 (*OsKS4*), Os10g0346300 (*Laccase-15*) and Os11g0641500 (*Laccase-19*) belonging to secondary metabolism were also more up-regulated in the AsIII treatment in comparison to AsIII + SNP treatment at both time intervals. Results showed that plants experienced more toxicity in the AsIII treatment in comparison to AsIII + SNP treatment (Figs 1 and 2). In general, up-regulation of genes in AsIII stress may be due to higher toxicity and might be a strategy to cope with higher AsIII toxicity. In the present study, we observed modulation in genes expression of terpene and lignin biosynthetic pathways such as Os02g0570400 (*OsTPS6*), Os02g0571100 (*OsTPS7*), Os04g0178300 (*OsTPS15*), Os04g0179700 (*OsTPS16*), Os09g0400400 (*OsCAD8D*) and OS02G0713900 (*OsHMGR1*) which were differentially expressed in the AsIII + SNP treatment at both time intervals. It indicated the prominent role of NO in the modulation of secondary metabolite gene expression in exposure to AsIII. The genes related to Ca signaling were found modulated, but most of them were annotated as uncharacterized. The Os01g0505600 (*OsCML11*) and Os02g0291400 (*OsCBL8*), related to calcium signaling were observed to be significantly up and down-regulated in the AsIII and AsIII + SNP treatments on 4^th^ day. The Os02g0291400 (*OsCBL8*) and Os10g0411500 (*OsSTA242*) genes were more down-regulated in the AsIII + SNP treatment than AsIII treatment on 12^th^ day. Plant activates their signaling cascade for tolerance and adaptation *vis-a-vis* heavy metal toxicity, and studies indicated that a complex crosstalk occurs between the different signaling pathways^51–54^. ROS also acted as a signaling molecule and activated MAP Kinase during lead stress^55^ and also showed the crosstalk among Ca, NO and modulation in enzyme activity during Cu stress^56^.

**Figure 8:**
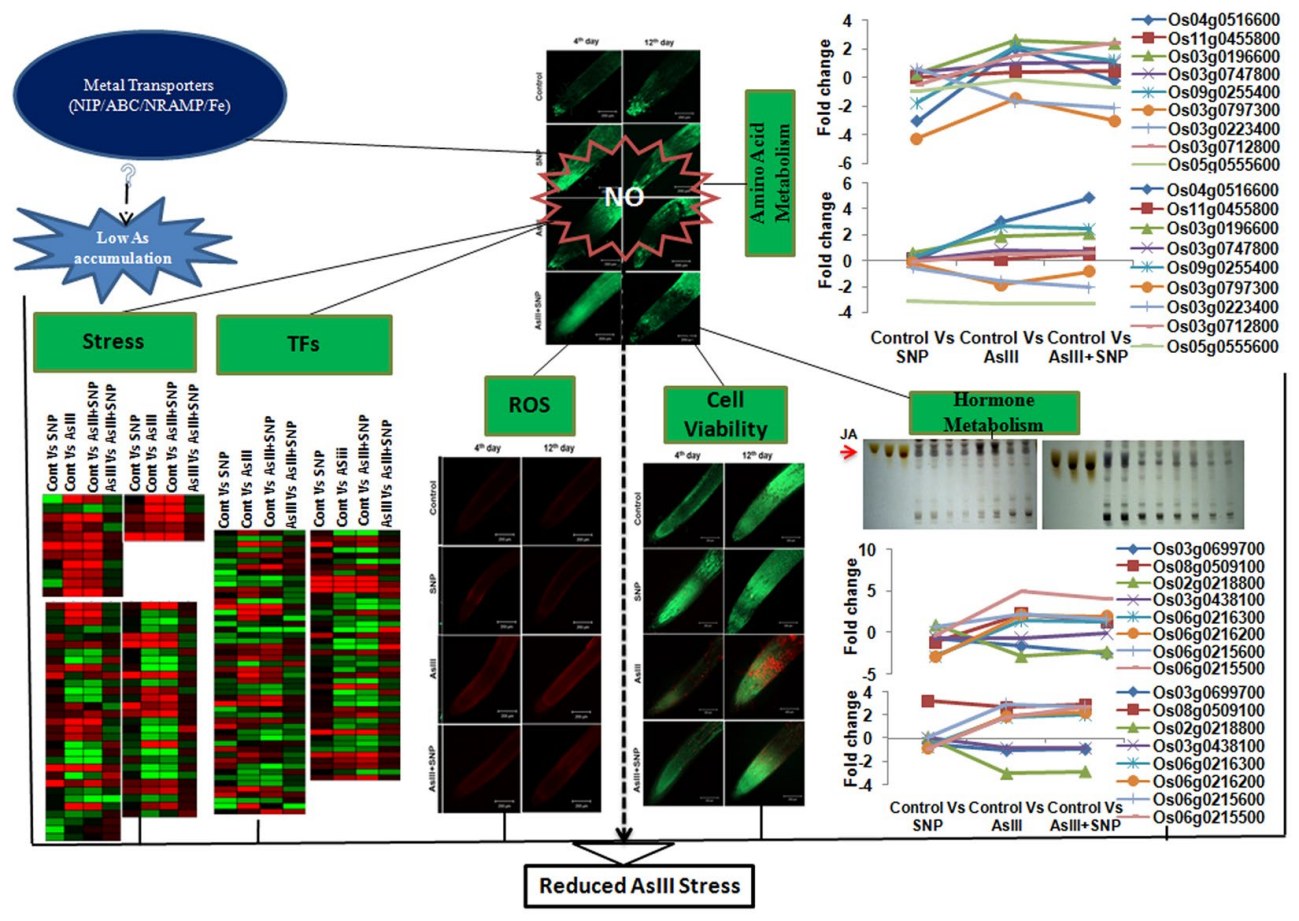
Schematic representation of NO-mediated AsIII stress detoxification mechanisms showing putative signaling among DEGs related to metal transport, stress response and several metabolic and cellular pathways in AsIII stress.

Recent studies indicated that NO had been known as a signal molecule in the induction of defense mechanisms in plants. Most abiotic stresses increased the *in planta* concentration of NO, which also points to its involvement in stress signaling. However, the molecular basis of NO function during arsenic toxicity is poorly understood. This transcriptomic study illustrated a molecular basis of NO response by modulating key regulatory pathways of AsIII stress (Fig. 8). SNP supplementation resulted in a reduction of As accumulation, suggesting the involvement of NO-responsive genes like NIP, NRAMP, ABC and iron transporters which might be of potential interest for further studies. This work also highlighted NO-mediated modulation of genes, especially TFs, CytP450, GSTs, GRXs, HSPs, amino acids and hormone(s) metabolism, indicating a potential role of NO in reduced ROS level, enhanced cell viability and root growth during AsIII stress. Reduction in jasmonic acid content following SNP supplementation further strengthens the JA-mediated protective role of NO during AsIII stress.

## Materials and Methods

### Experimental and Growth conditions

Seeds of rice genotype (*Oryza sativa* ssp. *indica* IC-115730) obtained from the National Bureau of Plant Genetic Resources, New Delhi, India, were used for this study. We used SNP as NO donor^46^. Several studies have been done to elucidate the biological role of NO by using SNP as NO donor in last few years^57–60^. Recently, Ou *et al*.^60^ studied the changes in DNA methylation in rice by using a high concentration of SNP (0.5 mM) as NO donor and also proved that cyanide or other by-product of SNP had no significant effect on rice plant at such higher concentration of SNP. Different concentrations of SNP (10 µM to 100 µM SNP) were screened in rice root under a hydroponic condition in half strength Hewitt media^61^ to find the best concentration at which root growth was enhanced (Supplementary Fig. 16). The maximum increase in root length (~17%) was observed at 30 µM SNP supplementation and selected for further experiments. Higher concentration of SNP inhibited root growth. The seeds were sterilized with 0.01% HgCl_2_ solution and washed with milli-Q water for germination in the dark (at ~26 °C for 4–6 days). Before treatments, germinated plantlets were grown in hydroponic medium (16/8 h; day/night, light intensity 210 µM m^−2^ s^−1^, 26/22 °C day/night temperatures; and 60% relative humidity) for 10 days and then exposed to AsIII (0.0, 25 µM, salt NaAsO_2_) supplemented with SNP (0.0, 30 µM SNP as NO donor) for 12 days. After the 4^th^ and 12^th^ day treatments, the plant root samples were washed with milli-Q water, immediately ground in liquid nitrogen and stored at −80 °C for further use.

### Arsenic estimation and quality control

Dried rice root samples (0.5 g) were crushed and digested in 3 ml of HNO_3_ at 120 °C for 2 h and then 80 °C for 4 h^62^. Total As was quantified by inductively coupled plasma mass spectrometer (ICP-MS, Agilent 7500 cx).

### RNA isolation and quality control

Frozen samples were ground in liquid nitrogen and made into a fine powder. RNeasy Plant Mini Kit (QIAGEN, MD) was used to isolate total RNA from root samples, following the manufacturer’s protocol. Total RNA was isolated in triplicate of each independent treatment. Independent triplicate of each treatment, which had 260/280 ratio ~2 and RIN value more than 8.5, were pooled and used for sequencing.

### Illumina sequencing, Read Mapping and Gene Expression Analysis

Illumina HiSeq procedure was followed for cDNA library preparations. Prepared libraries were run on Illumina HiSeq 2000 sequencing platform to generate the 100 bp paired-end reads. Low quality and adapter sequences were removed from the trimmed paired-end reads with the help of Bowtie2 (version 2.1.0). Pre-processed reads were used for reference based pair-wise alignment. The reads were aligned against reference rice genome, and gene model downloaded from Ensembl (http://plants.ensembl.org/Oryza_sativa/Info/Index) [Release-26] using Tophat program (version 2.0.8)^63^ with default parameters. Uniquely aligned reads were used for estimating expression of the genes and transcripts using Cufflinks program (version 2.0.2)^64^. Differential expression analysis was performed using Cuffdiff program (version 2.0.2). Summarized bioinformatics analysis pipeline is given in Supplementary Fig. 6A.

### PCA, Hierarchical clustering and Circos analysis

In order to analyze global expression patterns of different samples, PCA was carried out using Statistica 7 and Sigma Plot. Hierarchical clustering was done using pvclust package (https://cran.r-project.org/web/packages/pvclust/index.html) with default settings with Pearsons correlation coefficient^65^. Circos analysis was performed using Circos 0.66 software (<300 FPKM).

### Gene annotation, heatmap of differentially expressed genes and K-means clustering

Differentially expressed genes were annotated using PageMan^66^. Heat map of selected differentially expressed genes and K-means clustering were performed with the help of MeV 4.9 (http://sourceforge.net/projects/mev-tm4/files/mev-tm4/). The different sets of genes for K-means clustering were selected on the basis of significance level p ≤ 0.05.

### Quantitative real-time PCR

5 µg of total RNA was used to synthesize First cDNA strand using RevertAid First Strand cDNA Synthesis Kit (Thermo Scientific Molecular Biology), following the manufacturer’s protocol. The qRT-PCR primer pairs were designed by using Prime3Plus online software http://www.bioinformatics.nl/cgi-bin/primer3plus/primer3plus.cgi (Supplementary Table 9). StepOnePlus real-time PCR System (Applied Biosystems, USA) was used to perform the reactions, an initial 95 °C for 20 s, followed by 40 cycles of 95 °C for 3 s, 60 °C for 30 s in 96 well plate. Rice actin gene primers were used as an internal control. We also identified additional reference genes (Os01g0610100) from our data using Normfinder to validate the RNA-Seq data^67^. The delta-delta CT method was used to analyze the data^68^. The expression patterns of genes were matched with RNA-Seq data.

### Microscopic detection of nitric oxide (NO), superoxide radicals (O_2_^·−^) and cell viability

Nitric oxide, O_2_^·−^ and cell viability were detected in rice root of control and treatments (SNP, AsIII and AsIII + SNP) on 4^th^ and 12^th^ day. Fluorescent probe 4-aminomethyl-2′, 7′ difluorofluorescein diacetate (DAF-FM DA, Calbiochem) was used to detect NO in root^69^. Root was incubated in 10 µM DAF-FM DA (in 1x PBS, pH 7.2) for 30 minutes at 25 °C and washed thrice with PBS (for 5 minutes each) after staining^69^. Superoxide radicals (O_2_^·−^) were also detected using 10 µM dihydroethidium (DHE, Calbiochem)^70^. The microscopic observation was performed under a confocal microscope (LSM510 META, Carl Zeiss) with 10x Plan-Apochromat lenses using λex- 488 nm and λem- BP505–550 nm for NO detection and λex- 514 nm and λem- LP560 nm for superoxide radicals. Rice root cell viability was performed by incubating the root samples in the fluorescein diacetate (FDA, Sigma Aldrich, USA), propidium iodide (PI, Sigma-Aldrich, USA) for [2 mg/ml (FDA) in acetone was diluted by PBS drop by drop until the solution turned milky with 1 µg/ml PI (PBS) added to a final concentration] 5 minutes. The images were captured by using green (FDA; λex- 488 nm and λem- BP505–550 nm) and red channels (PI; λex- 543 nm and λem- LP560 nm) of the confocal microscope (LSM510 META, Carl Zeiss).

### Jasmonic acid (JA) measurement by HPTLC

Fresh rice root samples (5 g) were ground in liquid nitrogen, and 15 ml of extraction solvent (2-propenol: water: HCL; 2:1:0.002 vol./vol./vol.) was added to each sample and shaken for 30 minutes at 100 rpm (4 °C). After that, 15 ml of dichloromethane was added to each sample and shaken for 30 minutes at 100 rpm (4 °C). Lower phase of centrifuged samples (13,000 *g* at 4 °C for 5 minutes) were taken and dried by a vacuum centrifugal concentrator for ∼1 h and then dissolved in methanol for further use^71^. Jasmonic acid was measured by HPTLC (using Camag TLC Scanner 3) in prepared samples^72^. Different concentrations of JA [(Lane 1, 10 µg; Lane 2, 20 µg; Lane 3, 40 µg) Sigma-Aldrich, USA] were used as standards.

### Data access

The Illumina sequencing reads of all samples have been submitted to NCBI BIO PROJECT (PRJNA357668) submission no. SUB2179893.

### Statistical analysis

Student’s T-Test (p ≤ 0.05) and One Way ANOVA (SPSS 16.0 software) were used to analyze the significance level in samples.

## Electronic supplementary material

Nitric oxide mediated transcriptional modulation enhances plant adaptive responses to arsenic stress

Supplementary Table 1

Supplementary Table 2

Supplementary Table 3

Supplementary Table 4

Supplementary Table 5

Supplementary Table 6

Supplementary Table 7

Supplementary Table 8

Supplementary Table 9
