# Supplementary material for: Nitric oxide mediated transcriptional modulation enhances plant adaptive responses to arsenic stress
Source: Sci Rep. 2017 Jun 15;7:3592. doi: 10.1038/s41598-017-03923-2 (PMC5472597; doi:10.1038/s41598-017-03923-2)
Supplement: Supplementary file 1 — Nitric oxide mediated transcriptional modulation enhances plant adaptive responses to arsenic stress [file 41598_2017_3923_MOESM1_ESM.pdf]

Nitric oxide mediated transcriptional modulation enhances plant adaptive responses to arsenic stress

Pradyumna Kumar Singh[1,2], Yuvraj Indoliya[1,2], Abhisekh Singh Chauhan[1,2], Surendra Pratap Singh[1], Amit Pal Singh[1], Sanjay Dwivedi[1], Rudra Deo Tripathi[1,2*], Debasis Chakrabarty[1,2*]

[1] Council of Scientific and Industrial Research - National Botanical Research Institute (CSIR-NBRI), Rana Pratap Marg, Lucknow-226001, India

[2] Academy of Scientific and Innovative Research (AcSIR), Anusandhan Bhawan, 2 Rafi Marg, New Delhi-110 001, India

*** Corresponding author; tripathird@gmail.com, chakrabartyd@nbri.res.in**

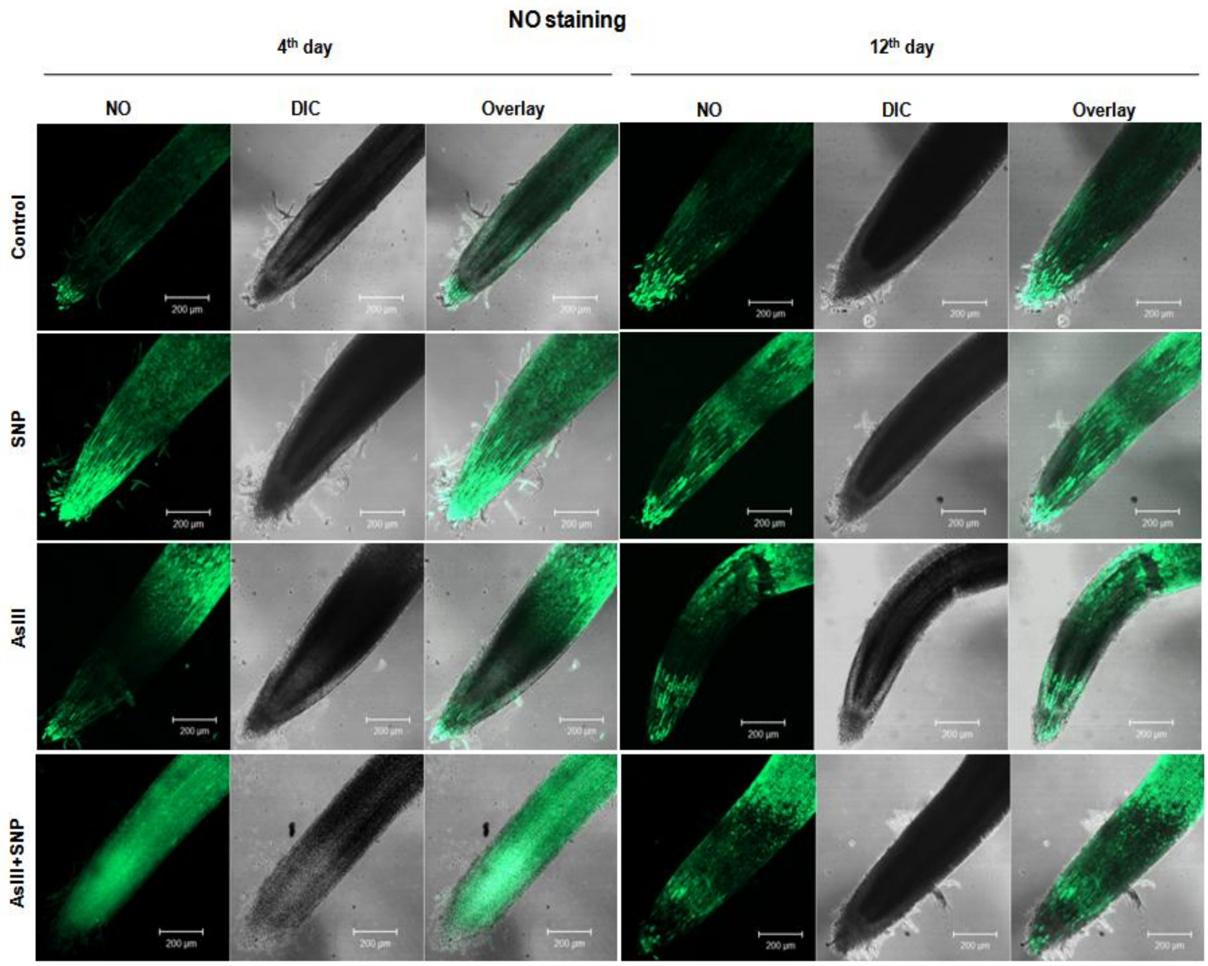

Supplementary Fig. 1: CLSM observation for NO on 4th and 12th day. DIC and overlay images of roots of NO observation in different treatment at both time intervals showed changes in NO level.

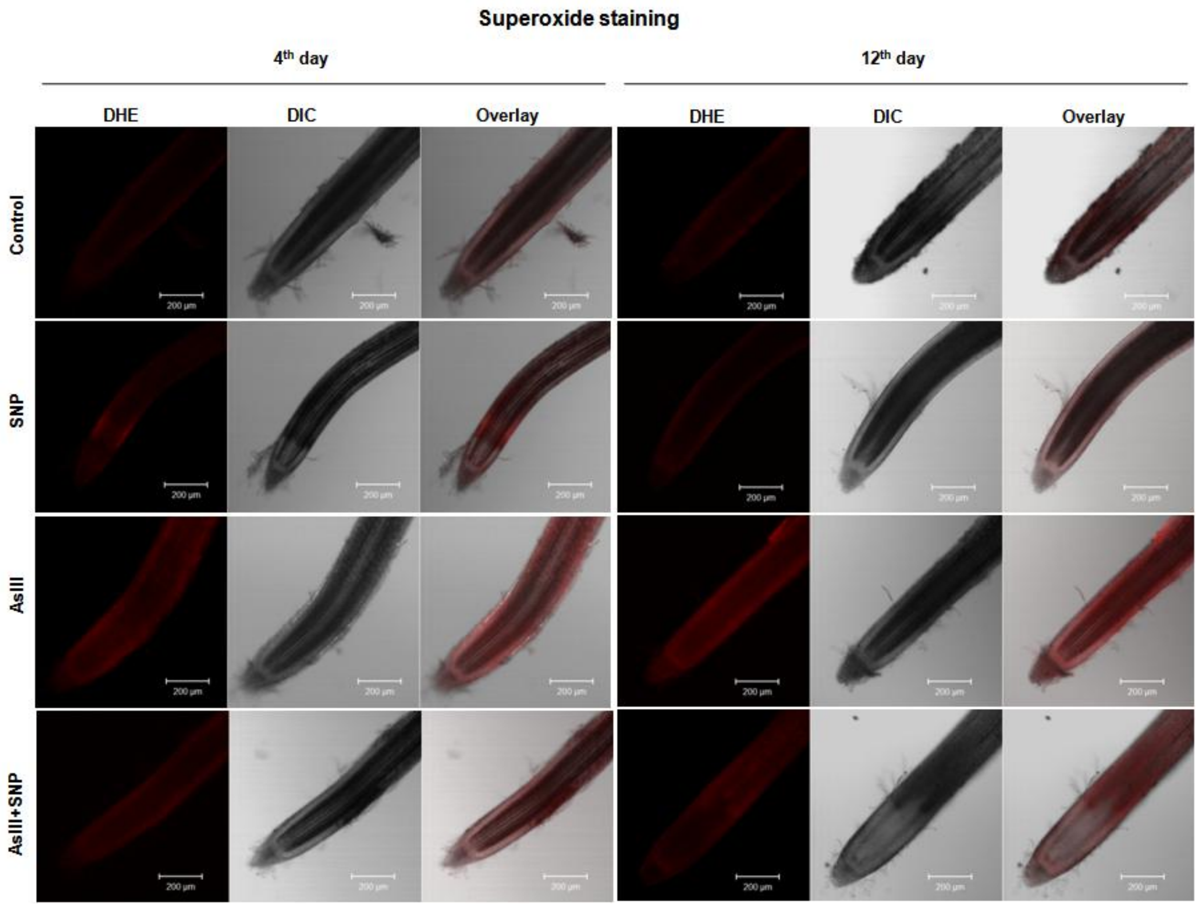

Supplementary Fig. 2: CLSM observation for superoxide on 4th and 12th day. DIC and overlay images of roots of superoxide in different treatment at both time intervals showed reduction in superoxide level in the AsIII+SNP treatment in comparison to AsIII treatment.

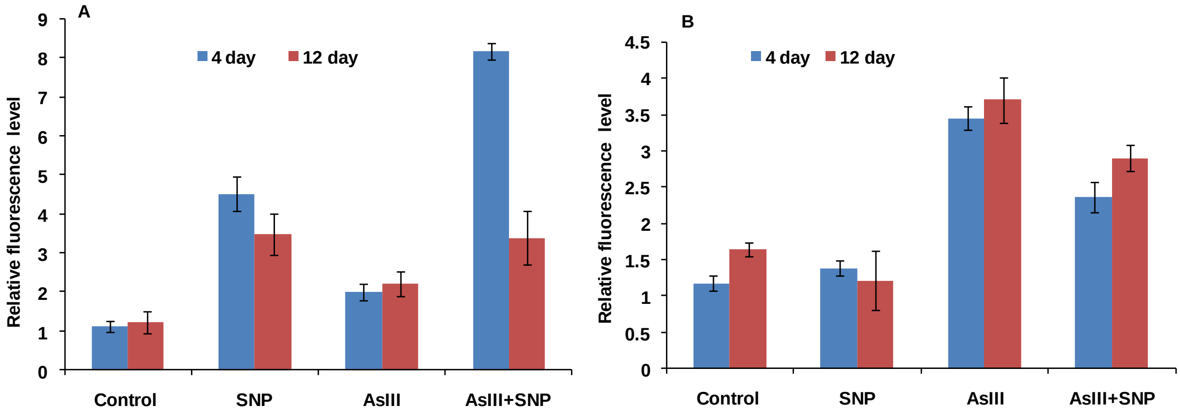

Supplementary Fig. 3: Relative fluorescence of NO (A) and superoxide (B) in different treatments at both time intervals. There is increase in NO level in the AsIII+SNP treatment in comparison to AsIII treatment while decrease in superoxide content in the AsIII+SNP treated root in comparison to AsIII treated root.

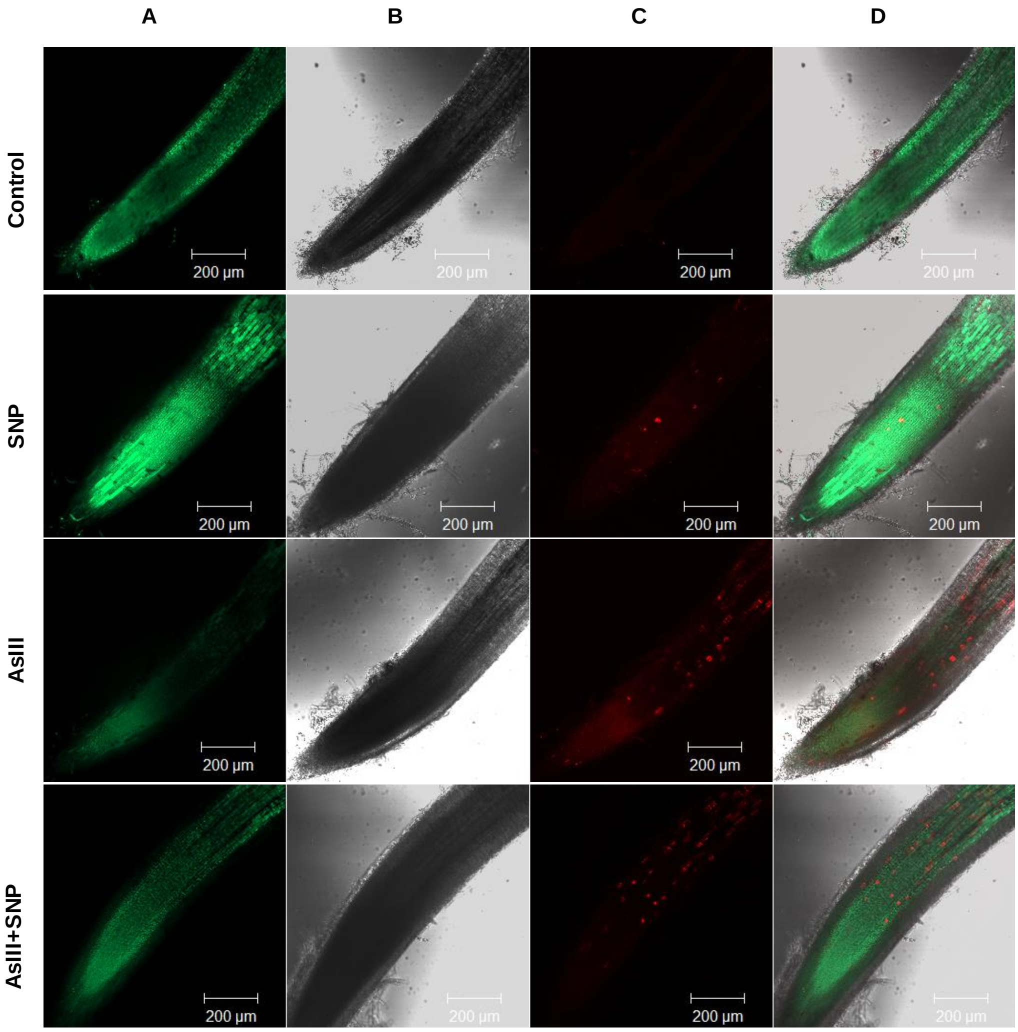

Supplementary Fig. 4: Images of cell viability assay on 4th day. Image of viable cell of root (A), DIC image of root (B), Image of dead cell of root (C) and overlay of all images (D), showed less toxicity in the AsIII+SNP treated root in comparison to AsIII treated root.

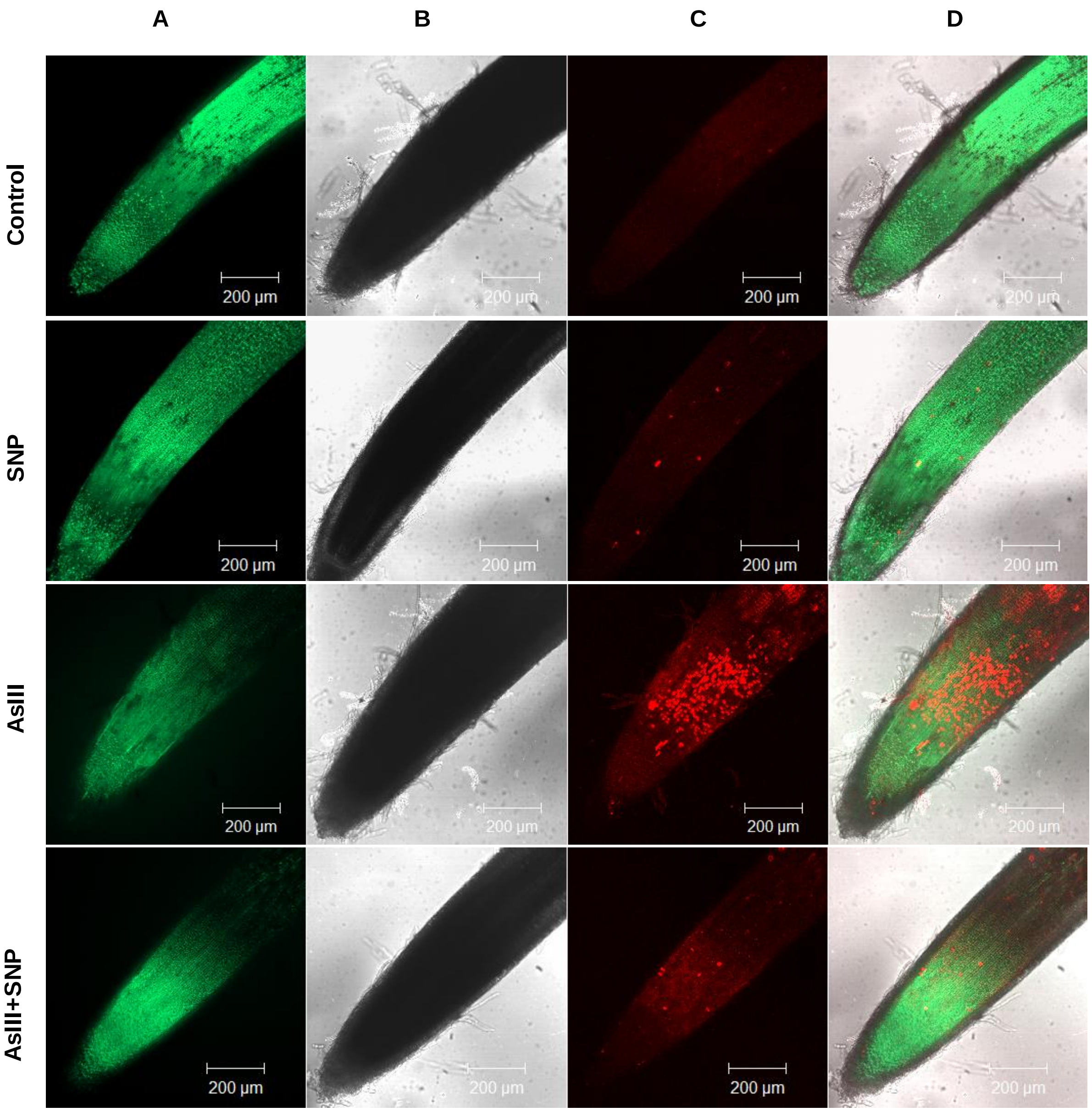

Supplementary Fig. 5: Images of cell viability assay on 12th day. Image of viable cell of root (A), DIC image of root (B), Image of dead cell of root (C) and overlay of all images (D), showed higher in the AsIII treatment in comparison to AsIII+SNP treatment.

A

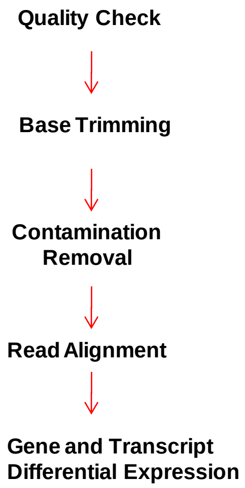

B

Sample (Root)	Total Reads (paired-end)	GC (%)	% of data>= Q30	Raw read length (bp)	Aligned Read Count	Aligned (%)
4 day Control	63,013,448	51.3	84.6	100 x 2	46,146,574	83.78%
4 day SNP	33,517,798	52.81	91.4	100 x 2	27,825,128	88.41%
4 day AsIII	125,737,228	50.48	86.3	100 x 2	99,229,580	83.84%
4 day AsIII+SNP	143,136,926	51.78	87.9	100 x 2	117,978,838	86.03%
12 day Control	85,587,594	53.01	83.6	100 x 2	56,991,320	84.71%
12 day SNP	38,545,688	53	90.2	100 x 2	25,971,729	68.49%
12 day AsIII	106,858,902	49.21	87.2	100 x 2	88,491,141	84.32%
12 day AsIII+SNP	69,586,772	49.27	89.2	100 x 2	49,301,534	76.08%

Supplementary Fig. 6: Bioinformatics pipeline for processed samples (A) and Summary of the fastq file (B).

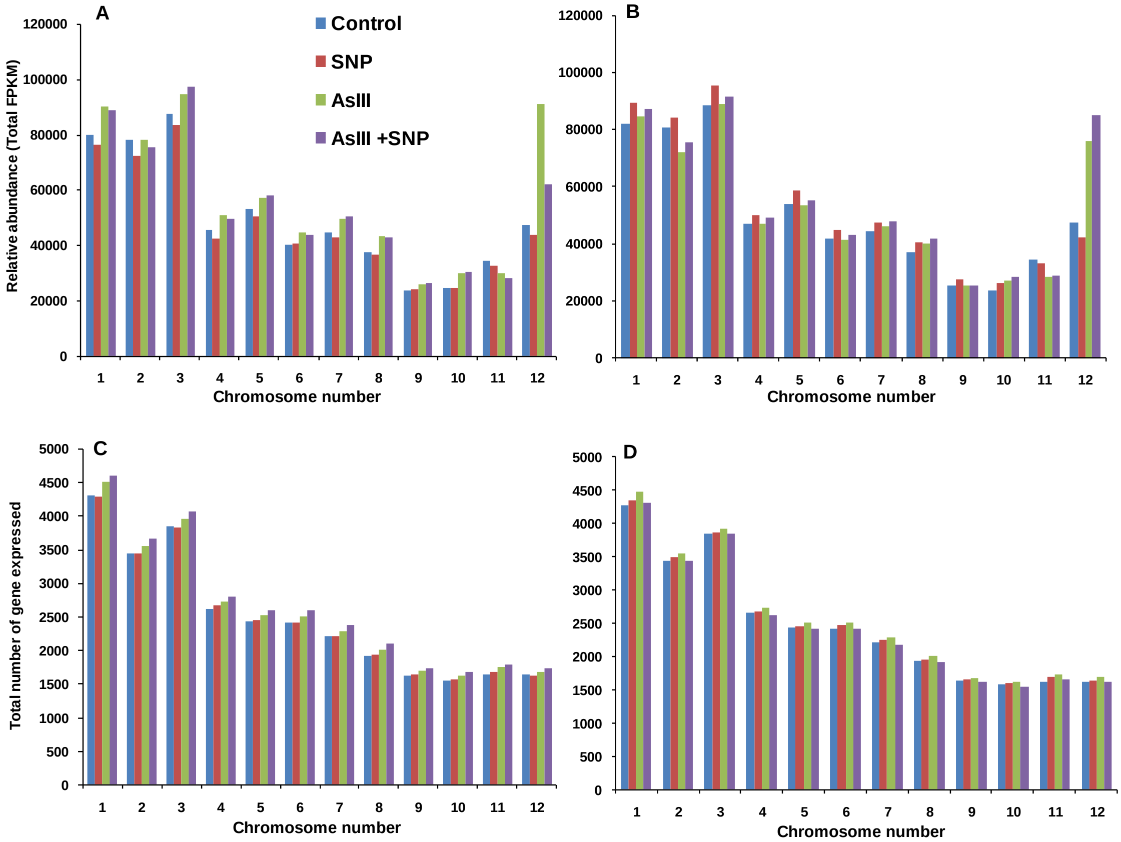

Supplementary Fig. 7: Relative abundance and total number of genes among different samples. A) Relative abundance (total FPKM) on 4th day, B) Relative abundance on 12th day, C) Total number of gene expressed per chromosome on 4th day, D) Total number of gene expressed per chromosome on 12th day.

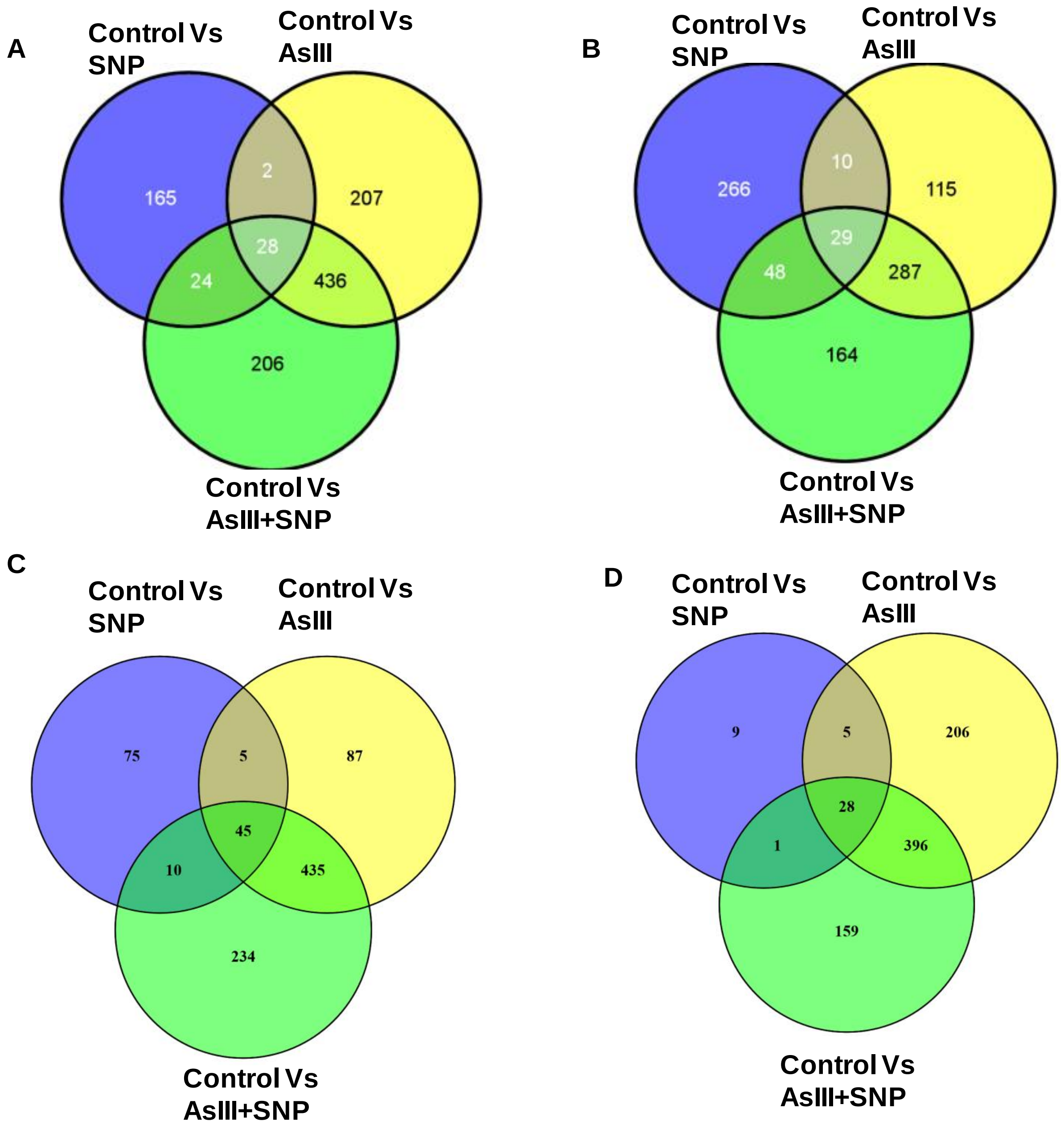

Supplementary Fig. 8: Analysis of up-regulated and down regulated differentially expressed ($p \leq 0.05$, fold change $\geq \pm \log 2$) genes by comparison between control and treated samples. A) Up-regulated genes and B) down-regulated genes on 4th day. C) Up-regulated genes and D) down-regulated genes on 12th day.

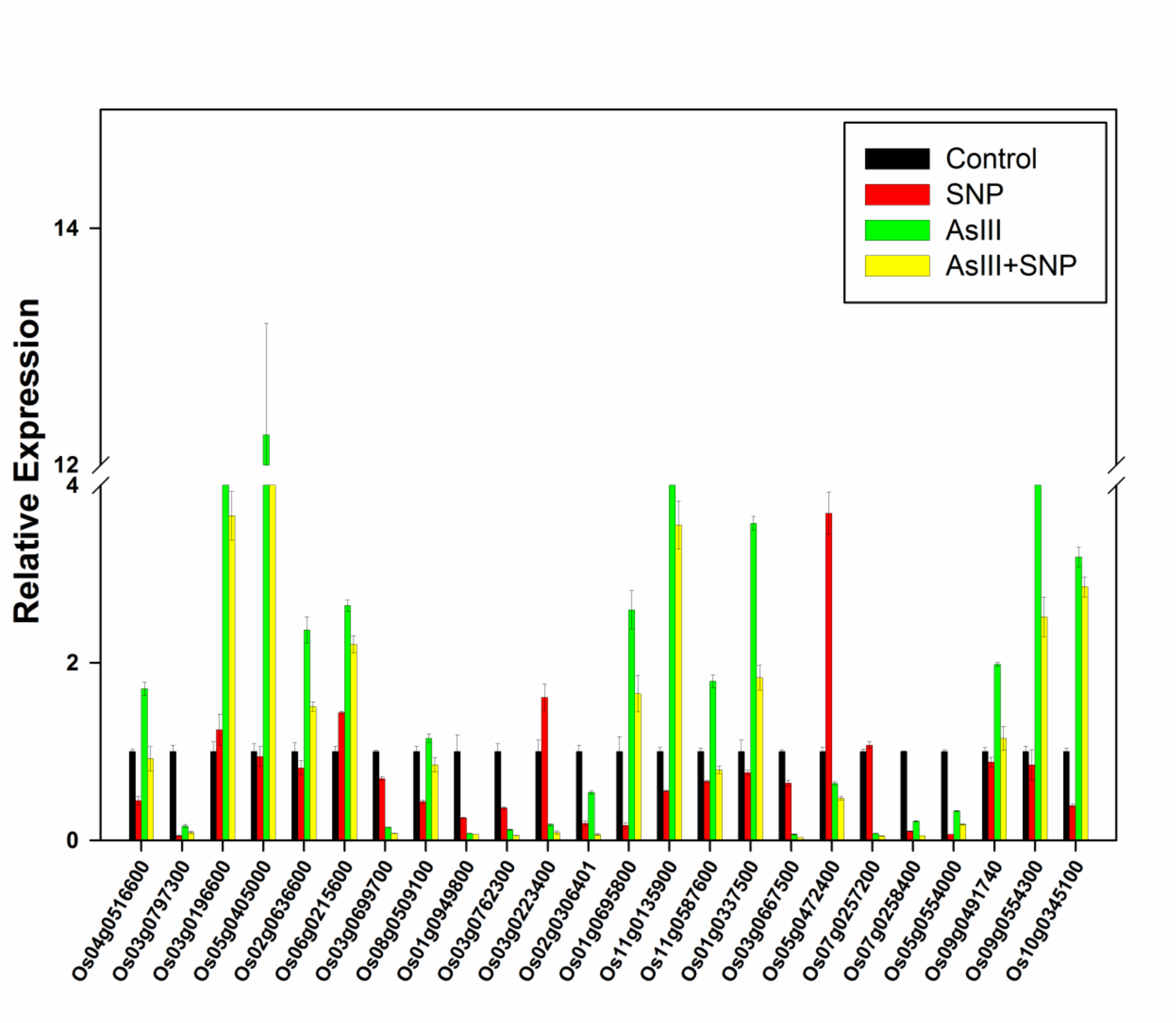

Supplementary Fig. 9: Validation of genes by real time PCR using actin as internal control on 4th day.

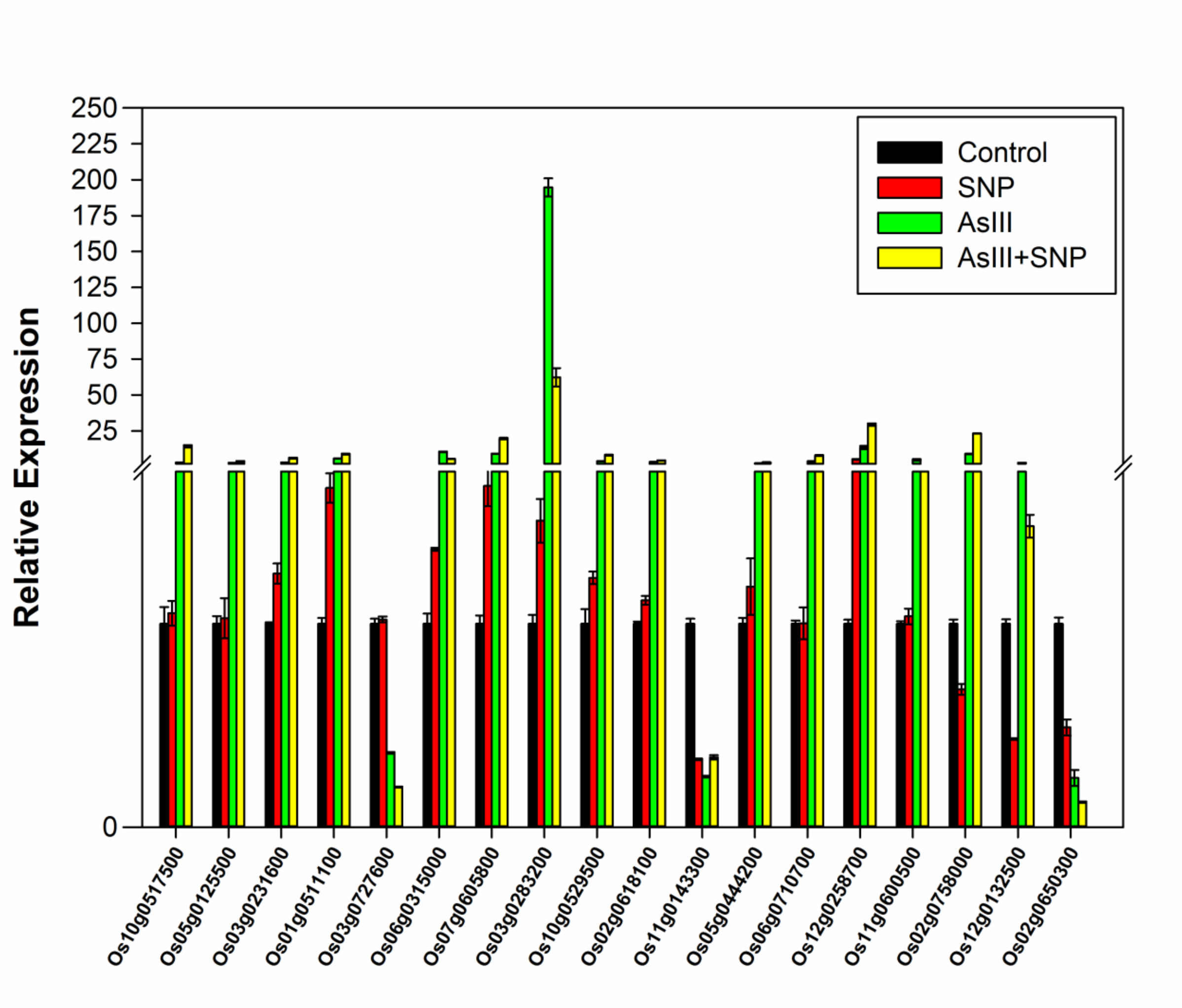

Supplementary Fig. 10: Validation of genes by real time PCR using actin as internal control on 12th day.

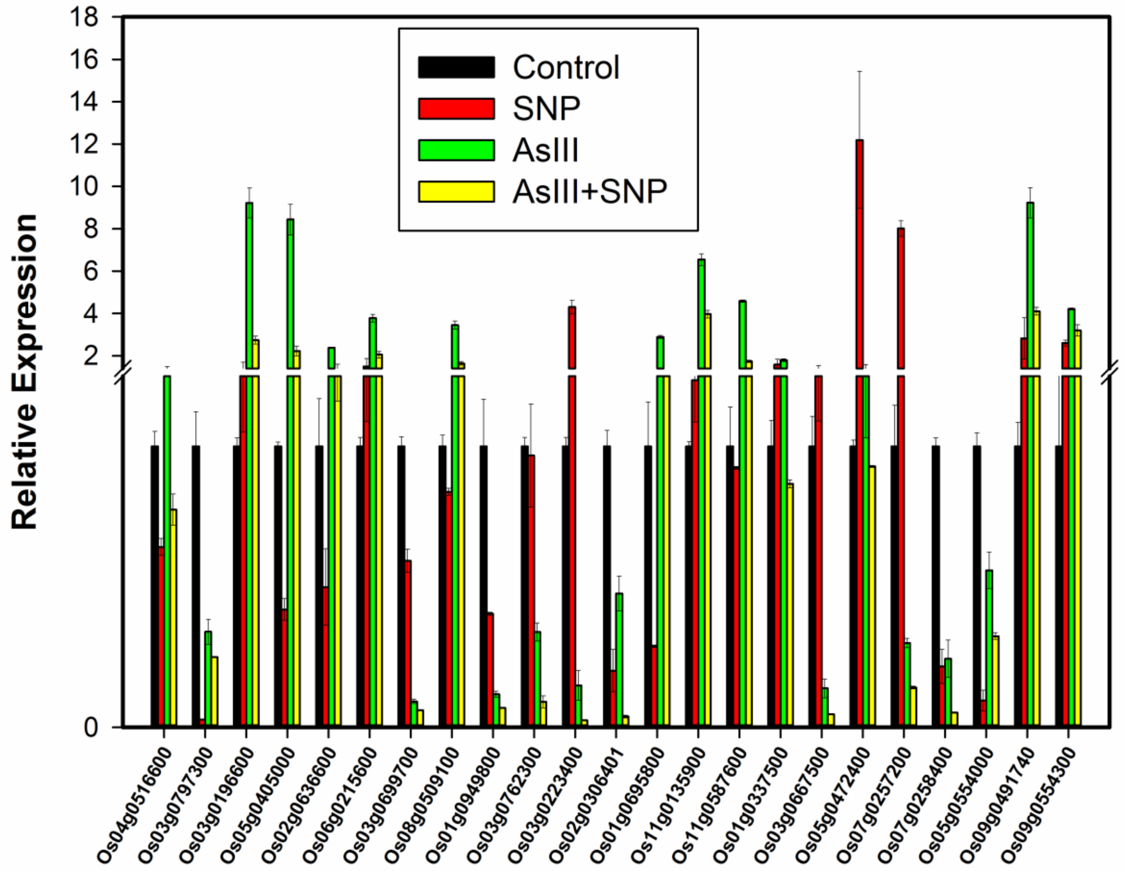

Supplementary Fig. 11: Validation of genes by real time PCR using Os01g0610100 as internal control on 4th day. The additional internal control Os01g0610100 was identified by using Normfinder (moma.dk/normfinder-software). The results showed similar expression patterns of genes with RNA-Seq data.

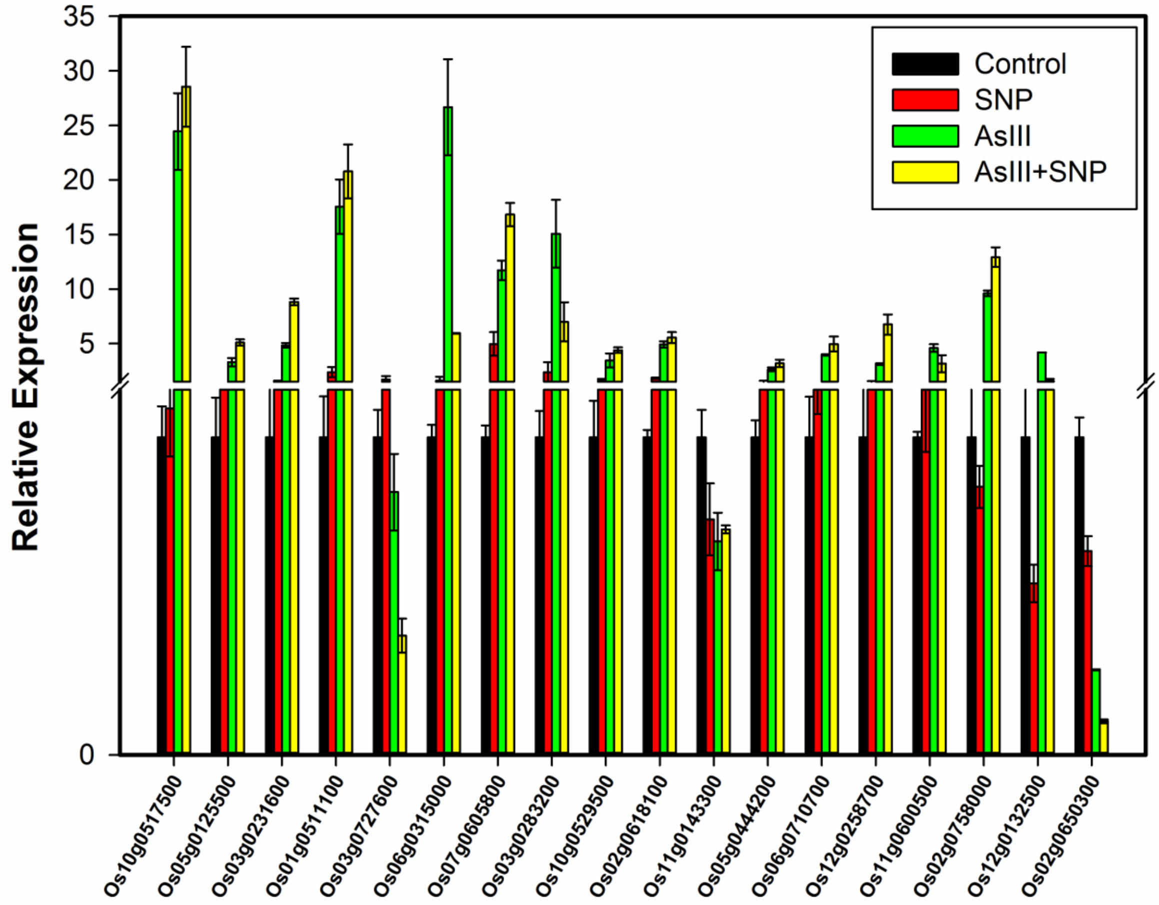

Supplementary Fig. 12: Validation of genes by real time PCR using Os01g0610100 as internal control on 12th day. The additional internal control Os01g0610100 was identified by using Normfinder (moma.dk/normfinder-software). The expression patterns of genes were matched with trancriptome data.

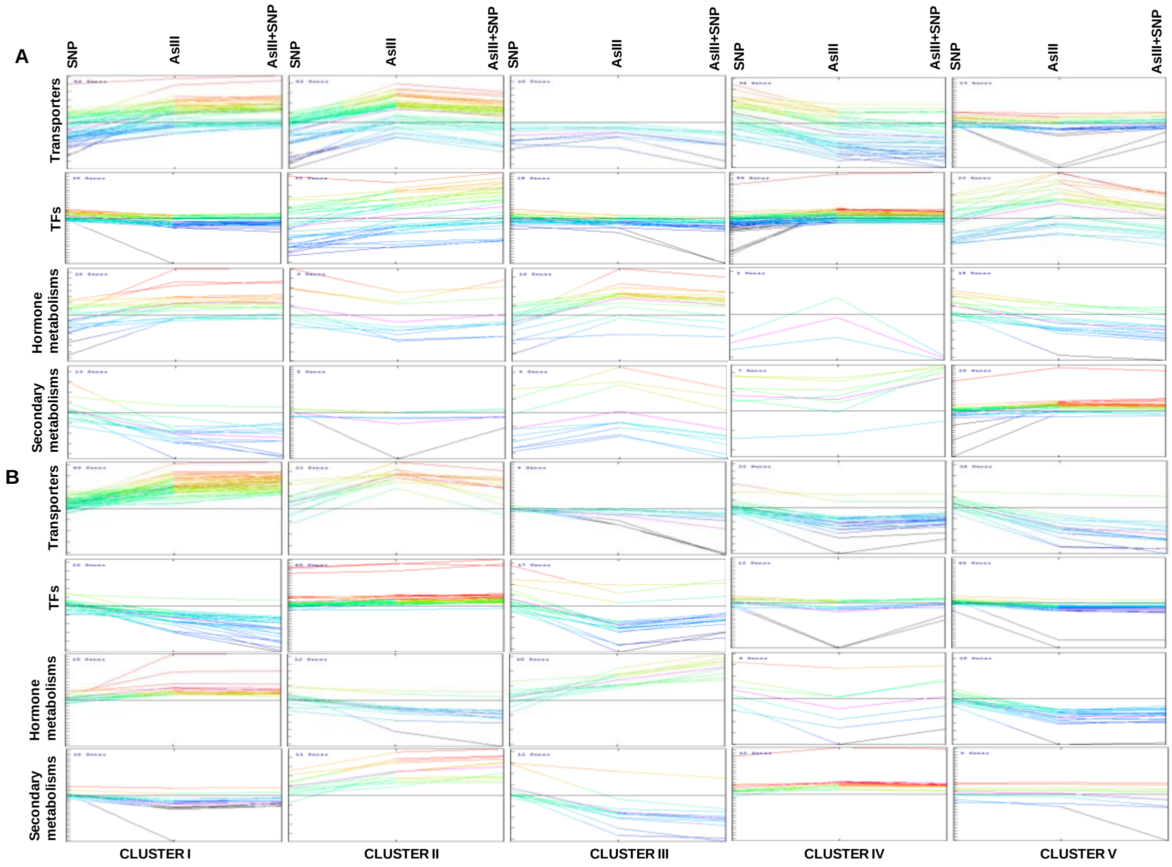

Supplementary Fig. 13: K-means clustering of different sets of genes on 4th day (A) and 12th day (B) showed expression behaviour of genes in categorized clusters.

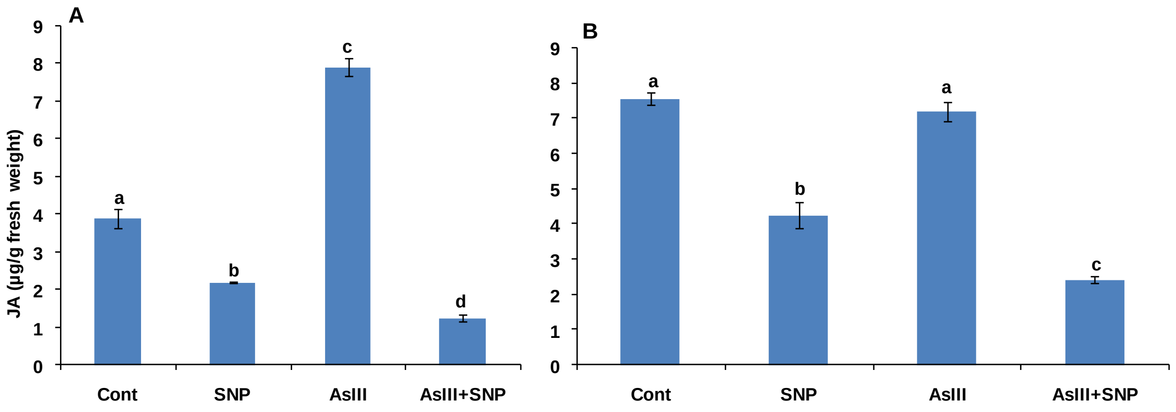

Supplementary Fig. 14: Quantification of JA content by HPTLC. The JA content on 4th day (A) and 12th day (B) showed reduction in JA level in the AsIII+SNP treatment in comparison to AsIII treatment. One Way ANOVA (SPSS 16.0 software) was used to analyze the significance level in samples.

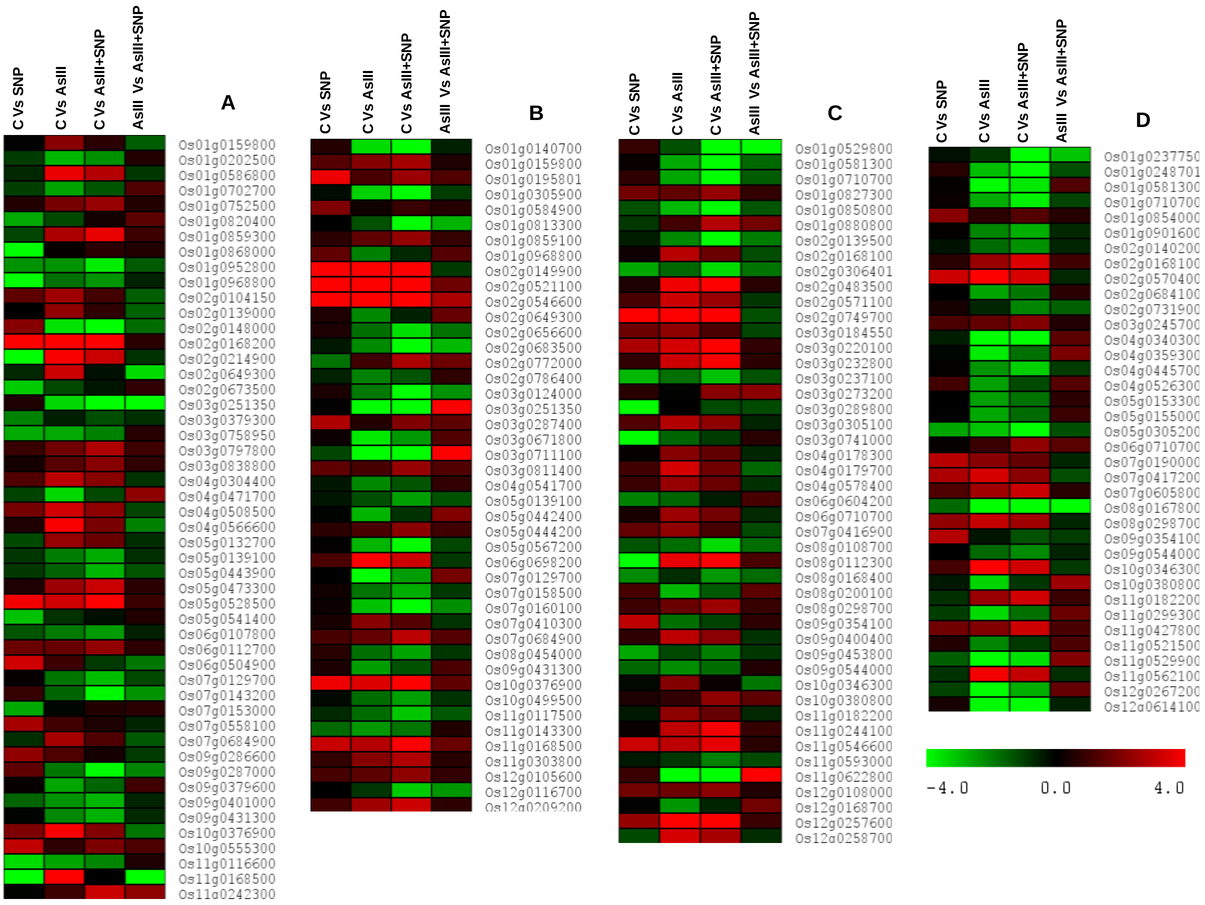

Supplementary Fig. 15: Differential expression analysis of genes of transcription factors (TFs), secondary and lipid metabolism. A) Represented genes expression of TFs on 4th day and B) represented genes expression of TFs on 12th day. C) and D) showed secondary and lipid metabolism genes on 4th and 12th day, respectively.

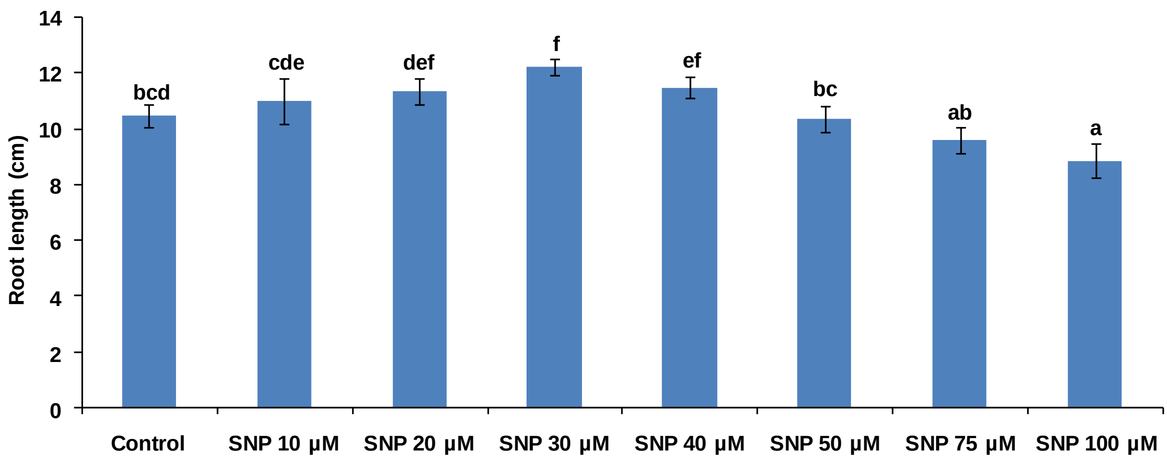

Supplementary Fig. 16: Selection of SNP concentration by using different concentration of SNP on 12th day showed maximum increase in 30 µM SNP treatment.
